# Electroconvulsive Therapy (ECT) Referral Workshop for Depression: Assessing Patients and Addressing Stigma

**DOI:** 10.15766/mep_2374-8265.11497

**Published:** 2025-02-11

**Authors:** Wayles Haynes, Jeremy Miller, Payton Weidner, Christopher Abbott

**Affiliations:** 1 Assistant Professor, Department of Psychiatry and Behavioral Health, University of New Mexico School of Medicine; 2 Associate Professor and Chief, Geriatric Psychiatry Division, Department of Psychiatry and Behavioral Health, University of New Mexico School of Medicine; 3 Second-Year Resident, Department of Psychiatry and Behavioral Health, University of New Mexico School of Medicine; 4 Professor and Chief, Neuromodulation Division, Department of Psychiatry and Behavioral Health, University of New Mexico School of Medicine

**Keywords:** Depression, Electroconvulsive Therapy (ECT), Geriatric Psychiatry, Neuromodulation, Case-Based Learning, Clinical Reasoning/Diagnostic Reasoning, Health Equity, Psychiatry, Diversity, Equity, Inclusion

## Abstract

**Introduction:**

Electroconvulsive therapy (ECT) is an important somatic treatment in psychiatry with well-defined indications, strong evidence for a quick response, and established efficacy. Despite the benefits of ECT, it is underutilized and inequitably accessed by patients in the United States. Patient and provider lack of knowledge, misinformation, and negative attitudes perpetuated by bias and stigma towards ECT can be significant barriers to patients receiving ECT.

**Methods:**

We created a workshop to address these issues with Kern's six-step approach, using the affective context learning theory as a conceptual framework. Based on a literature review and ECT physician researcher expertise, we taught the 90-minute workshop to 50 psychiatry and other behavioral health trainees to assess patients for ECT referral and address stigma and bias towards ECT. The workshop was delivered in person at four separate sessions to a total of 50 trainees and included pre- and postworkshop assessments of workshop efficacy.

**Results:**

Results of a paired-samples *t* test of participants’ responses pre- and postworkshop showed statistically significant growth on most learning objectives. Comments from the qualitative feedback indicated a majority-positive participant response to the engaging and effective workshop design and presentation. Participants’ self-assessment found at least a 50% normalized gain for all learning objectives.

**Discussion:**

This workshop is an effective contribution to ECT educational scholarship that can build trainee confidence in assessing patients with depression for ECT referral and addressing ECT stigma in clinical care.

## Educational Objectives

By the end of this activity, learners will be able to:

1. Assess patients with a depression diagnosis for electroconvulsive therapy (ECT) referral.
2. Describe the potential side effects, high-risk conditions, and alternatives for ECT.
3. Discuss how stigma towards ECT evolved.
4. Reflect on the impact of provider and patient stigma towards ECT.

## Introduction

Electroconvulsive therapy (ECT) is an efficacious and relatively fast-acting somatic treatment for depression that is clinically underutilized and inequitably accessed by patients.^1,2^ Multiple barriers exist for patients to receive ECT, including lack of patient knowledge or misinformation about ECT as well as negative attitudes perpetuated by societal stigma towards ECT.^3^ Provider attitudes that ECT is a treatment of last resort, lack of knowledge about indications for therapy, and fears about side effects of treatment can also perpetuate stigma and impair access.^4^ To address these issues, it is essential that both psychiatrists and other advanced practice providers are better trained to assess patients with depression for referral to ECT and to address stigma and bias toward ECT in clinical relationships.

Training psychiatrists and behavioral health providers in ECT has historically been challenging. A task force created in 2001 by the American Psychiatric Association (APA) to provide recommendations on treatment, training, and privileging for ECT found wide variation in US training programs’ ECT training.^5^ The task force on ECT recommended psychiatry residents should get at least 4 hours of didactic instruction on ECT, participate in at least 10 treatments, and assist in the care of at least three patients receiving ECT.^6^ ACGME program requirements for graduate medical education in psychiatry require competency in “indications for and uses of electroconvulsive and neuromodulation therapies,”^7^ but ACGME psychiatry milestones in somatic therapies lack specificity for ECT knowledge and skills.^8^ A 2008 study that surveyed 91 residency training programs found that resident ECT education varied considerably between programs, with most programs providing less than the APA Task Force recommendations and, at the same time, most believing ECT was underused nationally.^9^ Medical education scholars in Canada have developed specific ECT competencies to direct curriculum development and guidelines.^10^ However, we found no similar scholarship for ECT training in the United States. The overall variability and lack of data on current ECT education and the vague competencies and milestones required by credentialing bodies make it difficult to assess or guide trainee education in ECT.

Literature review of current ECT education resources found limited offerings. Intensive trainings for ECT certification are offered at some academic institutions, and the International Society for ECT and Neuromodulation offers a 1-day training course with companion online resources.^11^ Multiple textbooks on ECT exist, as well as various websites and video tutorials; however, these resources can be overly broad, are often designed for experienced ECT learners without innovative educational methods, and can lack the rigor of a scholarly, peer-reviewed work. No curricula specifically targeting ECT referrals for depression or on stigma towards ECT were found. *MedEdPORTAL* has no publications on ECT education, and for scholarship focused on neuromodulation therapies in general, one asynchronous learning module on transcranial magnetic stimulation exists.^12^

We created a workshop designed to engage emotions and explore stigma towards ECT through reflective discussions of patient stories in clinical care. Recent meta-analyses investigating the effectiveness of educational interventions to reduce stigma in mental health found significant reduction in learners’ stigma toward mental illness using lecture, case-based teaching, and role-play educational methods, with single- or multiple-session interventions equally effective.^13,14^ Utilizing these methods in this educational intervention, learners progressively build clinically relevant knowledge for ECT referrals and consider patient care situations significantly impacted by ECT stigma. This workshop begins to address the gap in ECT educational scholarship, is aligned with current ACGME somatic therapy competencies, and is the first publication in *MedEdPORTAL* for ECT assessment of patients and bias/stigma towards ECT.

## Methods

### Development

This workshop was created for psychiatry and other behavioral health trainees caring for patients with depression. Learners needed to have basic knowledge of depressive disorder diagnosis and management, including familiarity with efficacy and indications for ECT. Workshop facilitators had expertise with depressive disorder diagnosis, management, and referrals, as well as familiarity with the history and negative impacts of stigma towards ECT.

We applied the six-step Kern model when creating the workshop.^15^ For step 1—general needs assessment—we performed a literature review, identifying the gap in ECT education scholarship. We conducted interviews with local ECT research physicians, who reported a high need for training providers in ECT referrals due to the variable quality of referrals, barriers from patient and provider stigma/bias, and limited diversity of referred patients, which exacerbated disparities in ECT access. For step 2—targeted needs assessment—after completion of IRB review for the project (Human Research Review Committee ID# HRRCID 23-413), 23 of a possible 67 psychiatry department trainees at the University of New Mexico filled out emailed surveys assessing the perceived need for learning in identifying appropriate ECT referrals, describing stigma towards ECT, and discussing ECT as a therapy for patients, with 74%, 52%, and 78%, respectively, reporting a high to very high need for learning. For step 3—goals and learning objectives—we revised the original learning objectives for the workshop, using expertise from ECT research physicians and data from the needs assessment to narrow the workshop scope from all ECT diagnostic indications to focusing solely on depression, highlighting patient qualities that indicated the highest likelihood for successful treatment, and drawing from real-life examples of discrimination experienced by providers and patients for clinical vignettes. For step 4—educational strategies—we designed the workshop with the conceptual framework of affective context learning theory to foster learning with emotionally affecting educational experiences communicated through stories.^16^ A slide deck presentation introduced foundational knowledge through an interactive didactic interspersed with progressively complex case examples culminating in either a small-group discussion of a referral case or a role-playing activity to address stigma. Cases featured examples of patient presentations more and less likely to benefit from ECT as well as cases where clinical care was impaired by stigma towards ECT. Workshop facilitators intentionally led patient case discussions to engage the learners’ emotions and promote reflective practice focused on ECT care. For step 5—implementation—we delivered the first workshop in 60 minutes and the subsequent three workshops using a 90-minute time frame to psychiatry trainees in PGY 1 and PGY 2, PGY 3 and PGY 4, and PGY 5 and nurse practitioner trainees during required trainee didactic curriculum. For step 6—evaluation—workshop participants completed pre- and postworkshop REDCap surveys evaluating workshop efficacy through learner self-assessment of confidence in workshop objectives and using multiple-choice knowledge questions to target common misconceptions and mistakes found in ECT referrals.

### Implementation

The workshop was administered by one or two facilitators who spent a minimum of 1 hour reviewing course content and collaborating on roles before the workshop. The presenters used the facilitator guide (Appendix A) to lead the workshop with case and clinical script discussion points for group discussion. Suggested materials appropriate for conducting the workshop included audiovisual equipment for PowerPoint, pens, tables, and chairs arranged for small-group discussion. Suggested printed materials included copies of participant handouts (Appendix B), which featured the cases, small-group discussion questions, and role-play script for learners to utilize within the workshop, as well as evaluation forms (Appendix C) for pre- and postsurveys to assess learner knowledge and confidence in workshop objectives and to provide participants the opportunity to share what they liked or could be improved about the workshop.

The 90-minute workshop was performed using PowerPoint presentation (Appendix D), which served as the framework for the workshop. The first section discussed assessing patients with depression for referrals to ECT, utilizing successive case examples (30 minutes) and culminating in small-group discussion evaluating a case followed by a subsequent large-group discussion (15 minutes). The second section introduced the historical evolution of societal stigma towards ECT and issues with ECT stigma as a barrier to care (20 minutes) and culminated in a small-group reading of the ECT case example stigma script with reflection questions and follow-up large-group discussion (15 minutes). Remaining time (10 minutes) was utilized for questions and answers as well as the workshop evaluation (Appendix C).

We utilized a pre- and posttest research design to measure attitude (reaction) and knowledge (learning) levels of Kirkpatrick training evaluation. The evaluation tool was patterned after a previous *MedEdPORTAL* publication selected for its clarity and simplicity.^17^ The pre- and postworkshop surveys used multiple-choice questions to evaluate participants’ knowledge about indications for positive response to ECT, high-risk conditions for ECT, and ability to characterize possible cognitive side effects of ECT. The participants’ level of confidence with learning objectives before and after the workshop was assessed via a 5-point Likert-type scale (0 = *No Confidence,* 4 = *Completely Confident*). In the posttest, participants were asked to answer two open-ended questions: (1) “What did you like about this workshop?” and (2) “What suggestions do you have to improve this workshop?” Descriptive categorization of the comments by topic were determined by consensus between two of the coauthors (Wayles Haynes and Jeremy Miller).

## Results

The workshop was taught three times at the University of New Mexico and once at the Albuquerque Veterans Affairs center to 50 trainees. Of the 50 trainee participants, 42 (84%) completed the preworkshop evaluation, and 35 (70%) completed both pre- and postworkshop surveys. The workshop was facilitated by two presenters—a professor of geriatric psychiatry and ECT researcher—as well as a geriatric psychiatry fellow. Of the 35 respondents, two (6%) were advanced practice providers, 17 (49%) were PGY 1 and PGY 2 general psychiatry residents, seven (20%) were PGY 3 and PGY 4 general psychiatry residents, and nine (26%) were psychiatry fellows. The 35 respondents represented a diverse sample—19 (54%) identified as women; 15 (43%) as men; seven (20%) as lesbian, gay, bisexual, or queer; five (14%) as Asian; one (3%) as Black; eight (23%) as Hispanic/Latino; two (6%) as Middle Eastern; 19 (54%) as White; and 20 (57%) as nontraditional students.

In comparing responses pre- and postworkshop using paired-samples *t* tests (*n* = 35), we found a statistically significant increase in participants’ confidence to describe potential side effects, high-risk conditions, and alternatives to ECT (*p* < .01); to discuss how stigma towards ECT evolved (*p* < .01); and to reflect on provider and patient stigma towards ECT (*p* < .01). While the evaluation of confidence in assessing patients with depression for ECT did not reach statistical significance (*p* = .10), it did increase by 16%, with a normalized gain of 50%. Confidence results are exhibited in the Table. Participant responses to the knowledge questions about indications for positive response to ECT (*p* = .005), high-risk conditions for ECT (*p* = .08), and characterizing possible cognitive side effects of ECT (*p* > .99) showed variability.

**Table.**
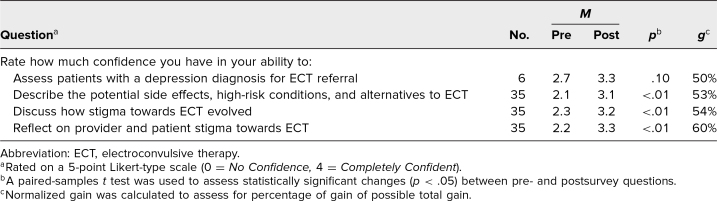
Pre- and Postworkshop Survey Responses on Participant Confidence

In the posttest, participants were given the opportunity to comment on two open-ended questions, “What did you like about this workshop?” and “What suggestions do you have to improve this workshop?” Comments for the workshop were overall positive, with a few suggestions for improvement. We organized the responses based on the most common topics addressing the quality of the workshop.

• Topic 1: The workshop content and presentation were engaging.
  (ECT) “The presentation of material was efficient without any loss of information. I also appreciate the amount of opportunities for engagement.”
  ○ “Engaging content, clear objectives, simple PowerPoint slides, and interaction with students—encouraging question.”
  ○ “Engaging, interactive, knowledgeable teacher who referenced many high-yield studies & clinical pearls. Made it fun.”
• Topic 2: The case-based and story driven workshop design was effective.
  ○ “Clear learning objectives, case-based application of principles, succinct, high value information.”
  ○ “Informative, memorable case presentations and discussions to illustrate key learning points.”
  ○ “Excellent use of patient vignettes in a way that engaged learners and supported material. Very effective at addressing educational goals. Clinically pertinent and useful information.”
  ○ “More didactics like this. Include it as part of standard lecture series.”
• Topic 3: Requested more time and detailed content.
  ○ “Longer time to deliver content would be nice.”
  ○ “Might benefit from a brief/concise mechanism of treatment/mechanism of therapeutic response to help contextualize how to explain the benefits to families.”

Overall, participants reported a well-designed and delivered workshop that fostered engagement in clinically relevant discussions on high-yield ECT topics. Despite missing data, survey responses found medium to large effect sizes in identifying learner confidence on most learning objectives and at least 50% normalized gains for all learning objectives, as well as significant improvement on two of the three knowledge questions. Participant comments expressed enthusiasm for story-based educational methods and interactive presentation style, supporting workshops designed in this effective and engaging style.

## Discussion

This workshop to educate psychiatrists and other behavioral health providers to assess patients with depression for ECT referral and to address stigma towards ECT is a successful education resource. The 90-minute workshop positively impacted participants’ confidence in addressing stigma and assessing patients. Participants’ feedback reported an engaged and effective workshop that provided clinically relevant learning in a fun and memorable style.

Several challenges arose during the workshop's development. The original scope, inspired by clinical need for improved referrals and equitable ECT access, was too broad. Workshop content was eliminated or realigned with revised learning objectives. The narrowed scope focused solely on ECT for depression and drew heavily on ECT research physician expertise for clinical cases. Deciding how to present ECT's evolution through psychiatry's history was also challenging. The dominant narrative, centered in the European-American worldview, rarely includes stories of people with more marginalized identities. We chose select events in ECT history to describe ECT stigma and acknowledge that future workshops could examine ECT's history from multiple diverse perspectives. An implementation challenge was completing the workshop content in the allotted 60 minutes. After feedback from the first delivery, workshop time was expanded to 90 minutes to accommodate all activities and discussions.

Project limitations include the short duration of intervention, single-site implementation, and simplistic evaluation design with incomplete participant response. Building capability in ECT referrals and addressing stigma toward ECT requires reinforcement, with clinical experiences, role modeling, and additional teaching beyond the brief intervention of this workshop. Due to the single-site implementation, it is unknown if the workshop would perform similarly at other locations and with different learners. The matched dataset pre-/posttest evaluation design has limitations such as having no comparison group or longitudinal assessment. To measure sustained change, participants could complete repeat surveys at a later point. Researchers could also track the quality of ECT referrals and the diversity of patients referred to ECT to measure behavior or clinical outcomes. Finally, workshop evaluation was challenging due to incomplete datasets impacting the power of the project and variable statistical significance. While participants’ increase in confidence for assessing patients with depression for ECT reached normalized gains of 50%, it was hindered by a small sample size (*n* = 6, *p* = .10). One possible reason that the knowledge question about cognitive impairment (*p* > .99) found no change was that was likely well-known foundational knowledge of behavioral health trainees.

Future iterations of this workshop could include improved evaluations and expanded scope. The workshop evaluations did not assess its impact on learner stigma towards ECT. Future workshop evaluations could assess learner stigma pre- and postworkshop to evaluate for change in trainee literacy, attitudes, and behaviors of stigma towards ECT. To more effectively assess knowledge gains on ECT stigma, the cognitive impairment question could be replaced with one on the historical development of stigma towards ECT. Other future directions of the work could include adapting the resources for asynchronous learning, diversifying the identities of patients and providers in case examples, validating clinical cases, and separating the two core workshop topics into separate workshops to increase the time allotted for learning and include additional educational methods.

Encouraging learners to address stigma and bias requires both knowledge to identify the issues and skills to mitigate the impact of these harmful forces. This workshop is only a first step in building a curriculum to enhance trainee capacity to care for patients with depression who might be experiencing barriers to care within inequitable systems. Future workshops might focus on other diagnoses or management concerns for patients receiving ECT, the history of somatic therapies in psychiatry, and the impact of health disparities on patients receiving ECT. Designing workshops with emotionally impactful learning experiences that shift learner attitudes might increase learner investment and competency in stigmatized treatments and marginalized patient populations. More medical education scholarship is needed to support training in ECT and to shape the future development of ECT educational guidelines and competencies. This workshop is the beginning of a high-quality, open-source, evidenced-based ECT curriculum.

## Appendices


Facilitator Guide.docxParticipant Handout.docxECT Referral Evaluation Form.docxECT Referral Presentation.pptx
*All appendices are peer reviewed as integral parts of the Original Publication.*
