# Supplementary material for: Electroconvulsive Therapy (ECT) Referral Workshop for Depression: Assessing Patients and Addressing Stigma
Source: MedEdPORTAL. 2025 Feb 11;21:11497. doi: 10.15766/mep_2374-8265.11497 (PMC11811188; doi:10.15766/mep_2374-8265.11497)
Supplement: Supplementary file 1 — Facilitator Guide.docxParticipant Handout.docxECT Referral Evaluation Form.docxECT Referral Presentation.pptx [file mep_2374-8265.11497-s001.zip › A. Facilitator Guide.docx]

Title: Electro-convulsive Therapy (ECT) Referral for Depression: Assessing Patients and Addressing Stigma

Overall Goal: This workshop will prepare learners to assess patients with a depression diagnosis for ECT referral and to address barriers of patient and provider stigma to accessing ECT.

Educational Objectives:

By the end of this activity, learners will be able to:

1. Assess patients with a depression diagnosis for ECT referral.
2. Describe the potential side effects, high risk conditions, and alternatives for ECT.
3. Discuss how stigma towards ECT evolved.
4. Reflect on provider and patient stigma towards ECT.

Resource Description and Intended Audience: This 90-minute interactive workshop utilizes case - based learning to teach behavioral health trainees (residents, nurse practitioners and physician assistants) to assess patients with depression for referral to ECT and to reflect on the impact of stigma towards ECT by both providers and patients. This workshop works best for audiences who already have some foundational knowledge about ECT efficacy, procedures, and recommended pathology. Cost to present workshop variable dependent on location resources.

| ECT Referral for Depression: Assessing Patients and Addressing Stigma | |
| --- | --- |
| Workshop Segment | Time |
| Introduction (Slides 1 – 4): pre-test, objectives, and basics of ECT care | 10 minutes |
| Didactic (slides 5 - 16):   - Who is likely to benefit from ECT? Indicators of response - When is an ECT referral appropriate? Timing and alternative therapies - What are the risks associated with ECT? Risks and side effects | 20 minutes |
| Case Based Learning (slide 17): Small group assessment of a patient presentation for ECT referral | 10 minutes |
| Large group discussion (slide 17): Facilitator guided discussion of learner responses | 5 minutes |
| Large group reflection (slide 18): Facilitator guided discussion of learner reflection on stigma and bias by providers and patients. | 5 minutes |
| Didactic (Slides 19 - 27)   - Introducing Stigma towards ECT with a brief history - Discussing Impact of stigma towards ECT on patient care - Addressing stigma towards ECT in patient provider relationships | 20 minutes |
| Partner Work (Slide 28): In pairs read through the provided script and answer the questions. | 10 minutes |
| Large Group Discussion (Slide 28): Share answers to questions in large group and ideas to implement in clinical life | 5 minutes |
| Objective Review, post workshop survey, resources (Slide 29 - 31) | 5 minutes |
| Total: | 90 minutes |

#### Required Resources

Common resources

1. PowerPoint Slides (Appendix D) with speaker notes
   1. When applicable, slides in the PowerPoint presentation contain speaker notes that provide the facilitator with a summary of the objective of the slide and example commentary.
2. Participant Handout (Appendix B) and Evaluation Form (Appendix C)
   1. To avoid printing, appendices could be available online with QR codes or links provided to learners to access them during class time (note required).
3. Ability to time individual and partner work sessions (either via wall clock, phone, etc.)

In-Person Version

1. Conference room with the following capabilities:

- 1. Seating for 25-30 participants
  2. Ability to project a PowerPoint presentation.

Virtual Version

1. Professional zoom account with ability to break groups up into break out rooms
2. Access to cloud share applications to use during small and large group discussions (Not required but recommended)
