# Supplementary material for: Electroconvulsive Therapy (ECT) Referral Workshop for Depression: Assessing Patients and Addressing Stigma
Source: MedEdPORTAL. 2025 Feb 11;21:11497. doi: 10.15766/mep_2374-8265.11497 (PMC11811188; doi:10.15766/mep_2374-8265.11497)
Supplement: Supplementary file 1 — Facilitator Guide.docxParticipant Handout.docxECT Referral Evaluation Form.docxECT Referral Presentation.pptx [file mep_2374-8265.11497-s001.zip › B. Participant Handout.docx]

The Case of Ms. Green: Transfer Request for ECT

Ms. Green is a 65-year-old female who was referred from an outside psychiatric hospital for ECT secondary to depression in the context of prolonged grief disorder. Her partner passed away from an opiate overdose 4 months ago. Ms. Green sees her deceased partner before bedtime most nights and finds it soothing to talk with him as she gets ready for bed. She’s getting worried that these hallucinations are “lasting too long”. She reports a low mood, anorexia without weight loss and a decrease in function with difficulty doing daily tasks including bathing and grooming. She is tearful most days and in pain because of a broken foot. Ms. Green is seeking ECT for her depression.

Outside hospitals record review from current hospitalization list diagnosis as unspecified depression with a concern for persistent grief disorder vs major depressive disorder with psychotic features. Ms. Green’s psychiatric history includes many years of waxing and waning depression with multiple medication trails including ketamine therapy without response. Her medical history is remarkable for a cardiac stent, controlled hypertension, and chronic back pain. Ms. Green fractured her foot one month ago. Current inpatient physician reports that she is ambulatory, the foot fracture is healing per repeat imaging, and the patient is on a scheduled pain management regime including substantial amounts of NSAID and opiates.

### Reflection questions: discuss and answer in small groups

1. Is Ms. Green an appropriate candidate for ECT referral? Support your answer with information from the case.
2. What are less favorable characteristics of Ms. Green’s presentation for ECT response?
3. What are more favorable characteristics of Ms. Green’s presentation for ECT response?

###

###

### The Case of Ms. Orange: Impact of Stigma on Care

Ms. Orange is a 53-year-old female long established with outpatient care who comes to an appointment with her psychiatrist Dr. Blue one week after discharge from an inpatient hospitalization. Her father, Mr. Orange, accompanies his daughter and was recently appointed her treatment guardian. Ms. Orange was admitted for a depressive episode due to concerns of worsening depressive symptoms, non-response to medications and grave passive neglect. Since returning to her family's home, she is still symptomatic with poor appetite, continued weight loss, low mood, is not showering or grooming, sleeps day and night, and has essentially stopped interacting with the family.

Ms. Orange is minimally conversant on interview, responding in all yes or no answers. She has a thin frame, is dressed in pajamas with poor grooming and hygiene. Her eyes are cast down and her body is hunched over with arms crossed in front. She is sad and questions the point of her life. “Nothing helps, I’ll never get better”. Her family is concerned, and her father wants help. Mr. Orange just finished telling Dr. Blue about his daughter’s condition.

Mr. Orange: My daughter is still very depressed. It’s the worst she’s been.

Dr. Blue: I think you are right to be concerned.

Mr. Orange: She’s worse now than when she went into that hospital, and they just let her go.

Dr. Blue: What therapy did she get during the last hospitalization?

Mr. Orange: Not much changed. They increased her medications but that just makes her sleep all the time. I don’t know how to help her.

Dr. Blue: I’m thinking that we may need to try something different than medications. Have you heard of ECT or electroconvulsive therapy?

Mr. Orange: Oh yes, the doctor in the last hospital told me it was a bad idea. That it shocks your brain and it’s barbaric. He thought it would be banned in 10 years.

Dr. Blue: That’s a strong statement. ECT does use electricity to induce a seizure but, ...

Mr. Orange: That’s what he said, a seizure. I don’t want to do that to her. She has enough issues with depression without having a seizure disorder too.

Dr. Blue: I think you’ve got some bad information. ECT just induces a seizure during treatment, it does not make you have seizures permanently. It’s relatively safe and effective even if some patients have memory issues.

Mr. Orange: Memory issues? She already has problems being slow. No, I don’t think so. That other doctor was a nice guy. I don’t think he’d lie to me.

Dr. Blue: He may not have lied, but he let his personal opinions impact your daughter's care. ECT is not barbaric. I have lots of patients who benefit from ECT. It’s been a very useful treatment when everything else fails. We are running out of options now. If you want your daughter to get better, she needs ECT.

Mr. Orange: I do want her to get better. That is why I’m here. That’s why I looked up ECT online. Lots of people say it’s torture. I don’t think ECT is a good idea.

Dr. Blue: Despite what you see on the internet, ECT is safe and effective. Let me show you some evidence….

Mr. Orange: No. We don’t want it. Please, what else do you recommend?

###

### Reflection Questions: Discuss and answer with a partner

1. How did stigma impact the care of Ms. Orange?
2. What issues arose in the conversation between Dr. Blue and Mr. Orange?
3. What reforming and reinforcing feedback would you give Dr. Blue as a provider addressing stigma towards ECT with patients and families?
