# Supplementary material for: Electroconvulsive Therapy (ECT) Referral Workshop for Depression: Assessing Patients and Addressing Stigma
Source: MedEdPORTAL. 2025 Feb 11;21:11497. doi: 10.15766/mep_2374-8265.11497 (PMC11811188; doi:10.15766/mep_2374-8265.11497)
Supplement: Supplementary file 1 — Facilitator Guide.docxParticipant Handout.docxECT Referral Evaluation Form.docxECT Referral Presentation.pptx [file mep_2374-8265.11497-s001.zip › C. ECT Referral Evaluation Form.docx]

**ECT Referral Workshop (HHRC ID# HRRCID 23-413): Pre-Test**

**Part I. Demographic Information**

**Select ONE circle that describes your current professional role:**

- Medical Student
- Medical Resident or Fellow
- Academic Faculty (MD, DO, NP, PA, Other)
- Clinician in Non-Academic Practice (MD, DO, NP, PA, Other)
- Other _________________
- I prefer not to answer.

**Please mark all that apply:**

- Female
- Male
- Transgender
- Gender Nonconforming
- Different Identity
- I prefer not to answer.

**Please mark all that apply:**

- Heterosexual or straight
- Gay
- Lesbian
- Bisexual
- Other ________________
- I prefer not to answer.

**Please mark all that apply:**

- Latino/Hispanic
- Puerto Rican
- Black/African American
- White
- Asian
- Native American/Alaskan or Pacific Islander
- Other ________________
- I prefer not to answer.

**Select ONE social class group that you most identify with:**

- Poor
- Working class
- Middle Class
- Affluent
- I prefer not to answer.

**Select ONE answer that describes you:**

- Traditional Student – consecutive school experience
- Non-traditional Student – break in school experience
- I prefer not to answer.

**Part II. Self-Assessment**

| **Please rate how much CONFIDENCE do you have in your ability to…** | **No**  **Confidence**  **0** | **Slightly Confident**  **1** | **Somewhat Confident**  **2** | **Fairly Confident**  **3** | **Completely**  **Confident**  **4** |
| --- | --- | --- | --- | --- | --- |
| Assess patients with a depression diagnosis for ECT referral. | 0 | 1 | 2 | 3 | 4 |
| Describe the potential side effects, high risk conditions, and alternatives for ECT. | 0 | 1 | 2 | 3 | 4 |
| Discuss how stigma towards ECT evolved. | 0 | 1 | 2 | 3 | 4 |
| Reflect on provider and patient stigma towards ECT. | 0 | 1 | 2 | 3 | 4 |

**Part III. Objective Assessment**

**Please Choose the Best Answer:**

1. The following is NOT a strong indicator of response to ECT?
   1. Episode duration of greater than 2 years.
   2. Greater severity of depressive symptoms
   3. Older age
   4. Prior response to ECT
2. What is a high-risk condition in patients for treating them with ECT?
   1. Pregnancy
   2. Diagnosis of Major Neurocognitive Disease, severe with behavioral disturbance
   3. Epilepsy with seizure within 6 months of ECT
   4. Myocardial infarction within 6 weeks of ECT
3. Cognitive impairment in ECT:
   1. Is permanent.
   2. If present will typically resolve within weeks.
   3. Is necessary for anti-depressant response.
   4. Is evidence of procedure mediated brain damage.

**ECT Referral Workshop Post-Test**

**Part I. Self-Assessment**

| **Please rate how much CONFIDENCE do you have in your ability to…** | **No**  **Confidence**  **0** | **Slightly Confident**  **1** | **Somewhat Confident**  **2** | **Fairly Confident**  **3** | **Completely**  **Confident**  **4** |
| --- | --- | --- | --- | --- | --- |
| Assess patients with a depression diagnosis for ECT referral. | 0 | 1 | 2 | 3 | 4 |
| Describe the potential side effects, high risk conditions, and alternatives for ECT. | 0 | 1 | 2 | 3 | 4 |
| Discuss how stigma towards ECT evolved. | 0 | 1 | 2 | 3 | 4 |
| Reflect on provider and patient stigma towards ECT. | 0 | 1 | 2 | 3 | 4 |

**Part III. Objective Assessment**

**Please Choose the Best Answer:**

1. The following is NOT a strong indicator of response to ECT?
   1. Episode duration of greater than 2 years.
   2. Greater severity of depressive symptoms
   3. Older age
   4. Prior response to ECT
2. What is a high-risk condition in patients for treating them with ECT?
   1. Pregnancy
   2. Diagnosis of Major Neurocognitive Disease, severe with behavioral disturbance
   3. Epilepsy with seizure within 6 months of ECT
   4. Myocardial infarction within 6 weeks of ECT
3. Cognitive impairment in ECT:
   1. Is permanent.
   2. If present will typically resolve within weeks.
   3. Is necessary for anti-depressant response.
   4. Is evidence of procedure mediated brain damage.

**Part III. Comments**

**Please answer the following questions:**

1. What did you like about this workshop?
2. What suggestions do you have to improve this workshop?
