# Supplementary material for: Electroconvulsive Therapy (ECT) Referral Workshop for Depression: Assessing Patients and Addressing Stigma
Source: MedEdPORTAL. 2025 Feb 11;21:11497. doi: 10.15766/mep_2374-8265.11497 (PMC11811188; doi:10.15766/mep_2374-8265.11497)
Supplement: Supplementary file 1 — Facilitator Guide.docxParticipant Handout.docxECT Referral Evaluation Form.docxECT Referral Presentation.pptx [file mep_2374-8265.11497-s001.zip › D. ECT Referral Presentation.pptx]

## Slide 1
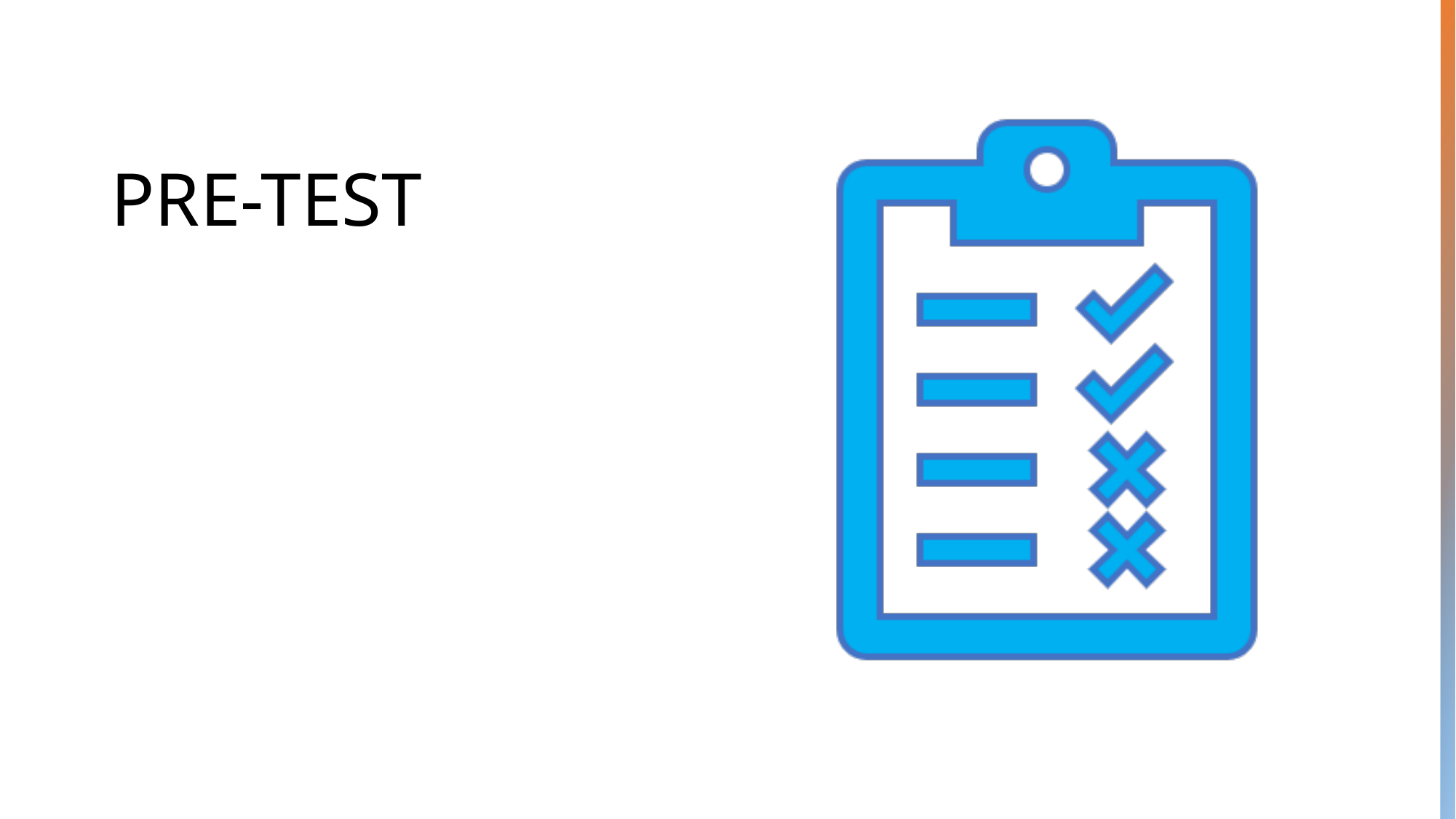

# PRE-TEST

## Slide 2
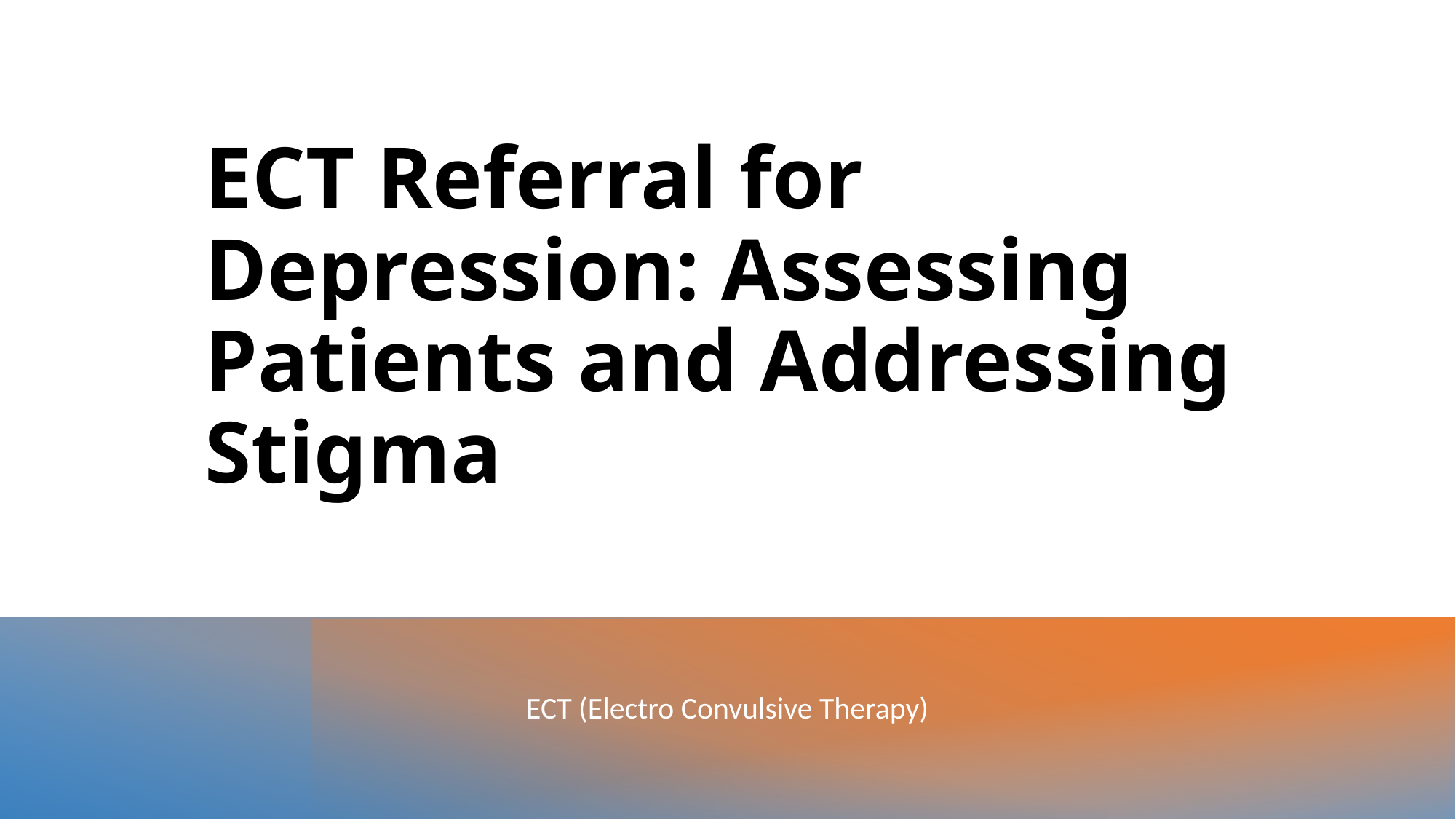

# ECT Referral for Depression: Assessing Patients and Addressing Stigma
ECT (Electro Convulsive Therapy)

## Slide 3
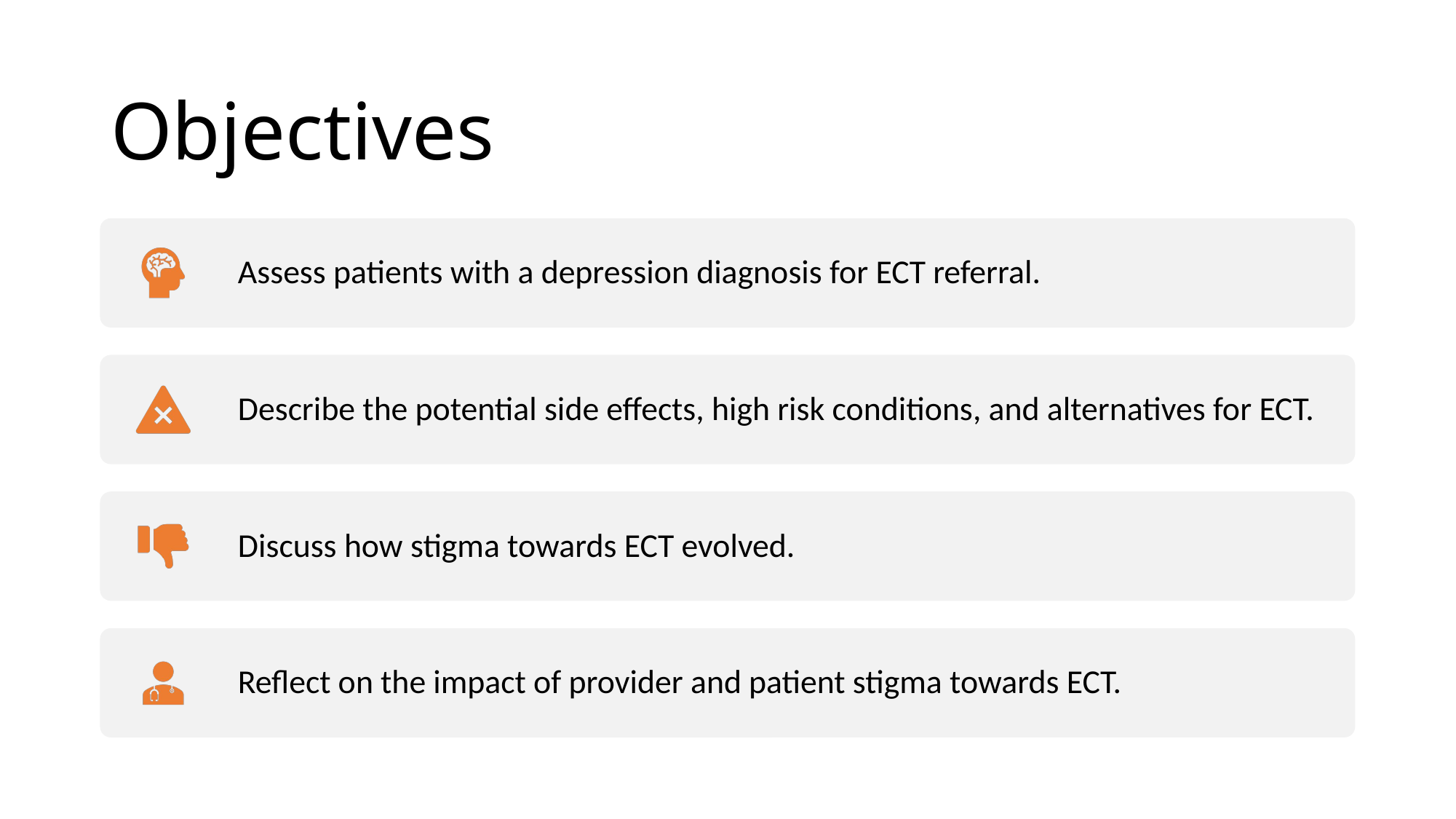

# Objectives

## Slide 4
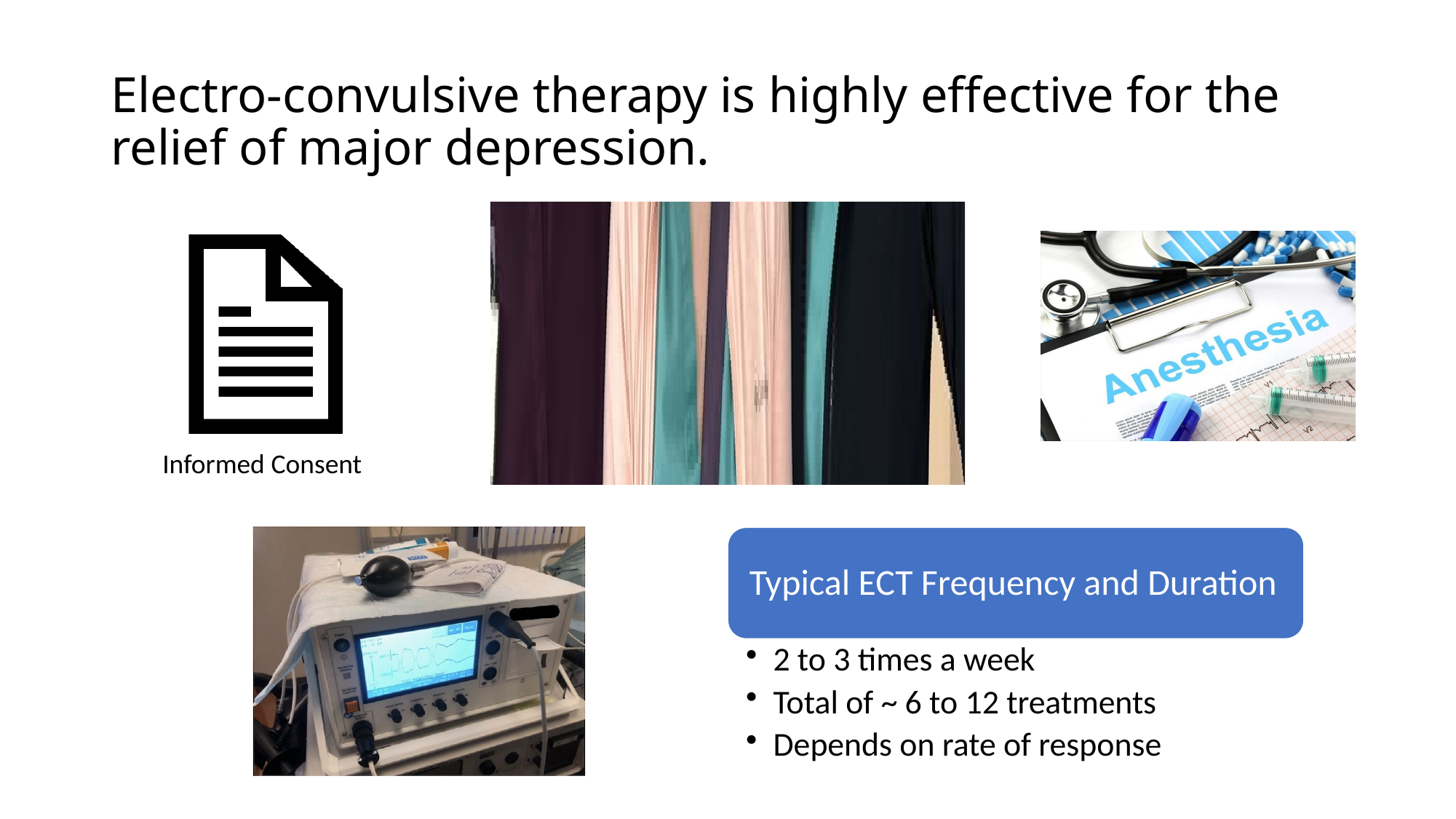

# Electro-convulsive therapy is highly effective for the relief of major depression.
Informed Consent

## Slide 5
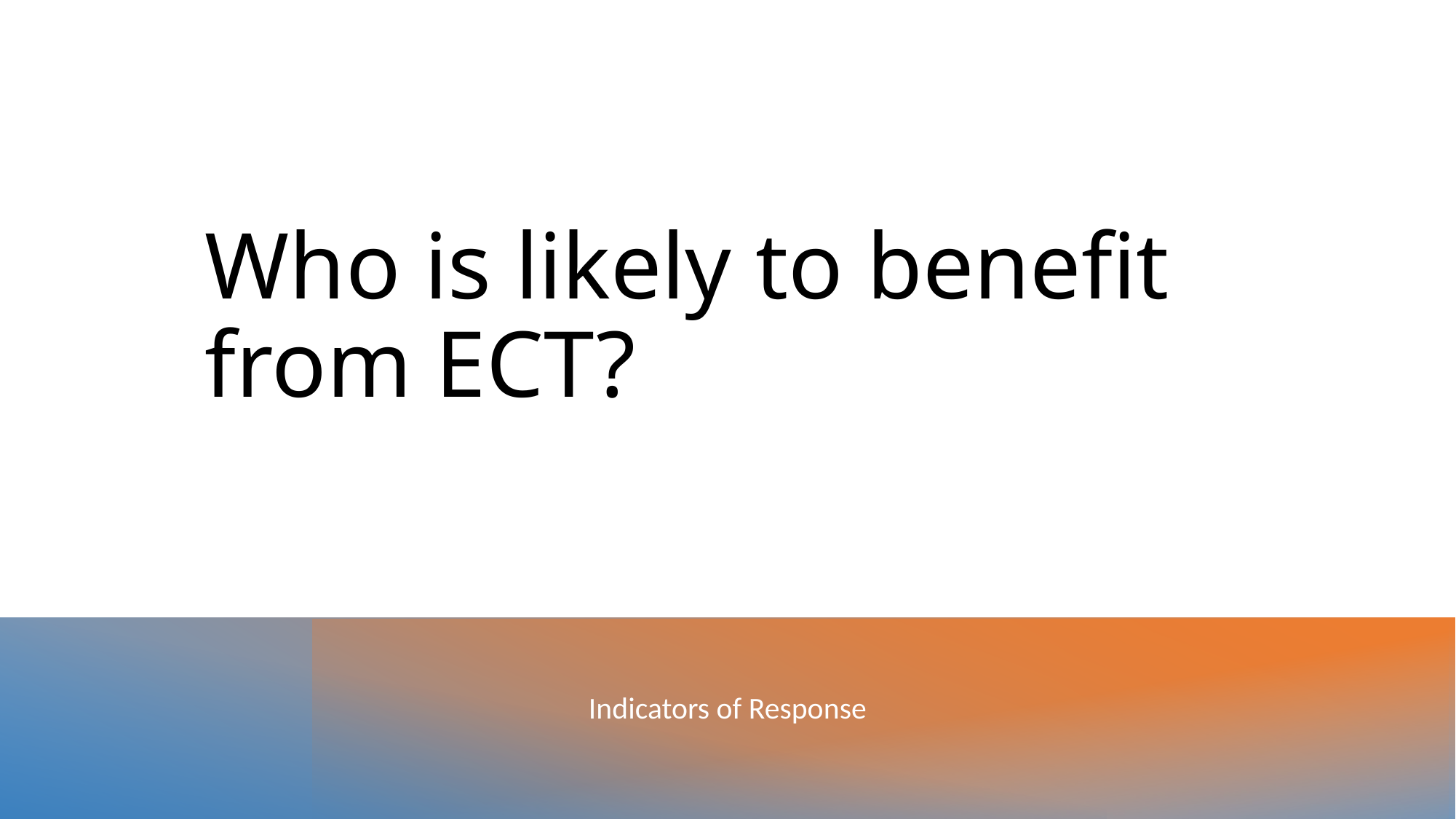

# Who is likely to benefit from ECT?
Indicators of Response

## Slide 6
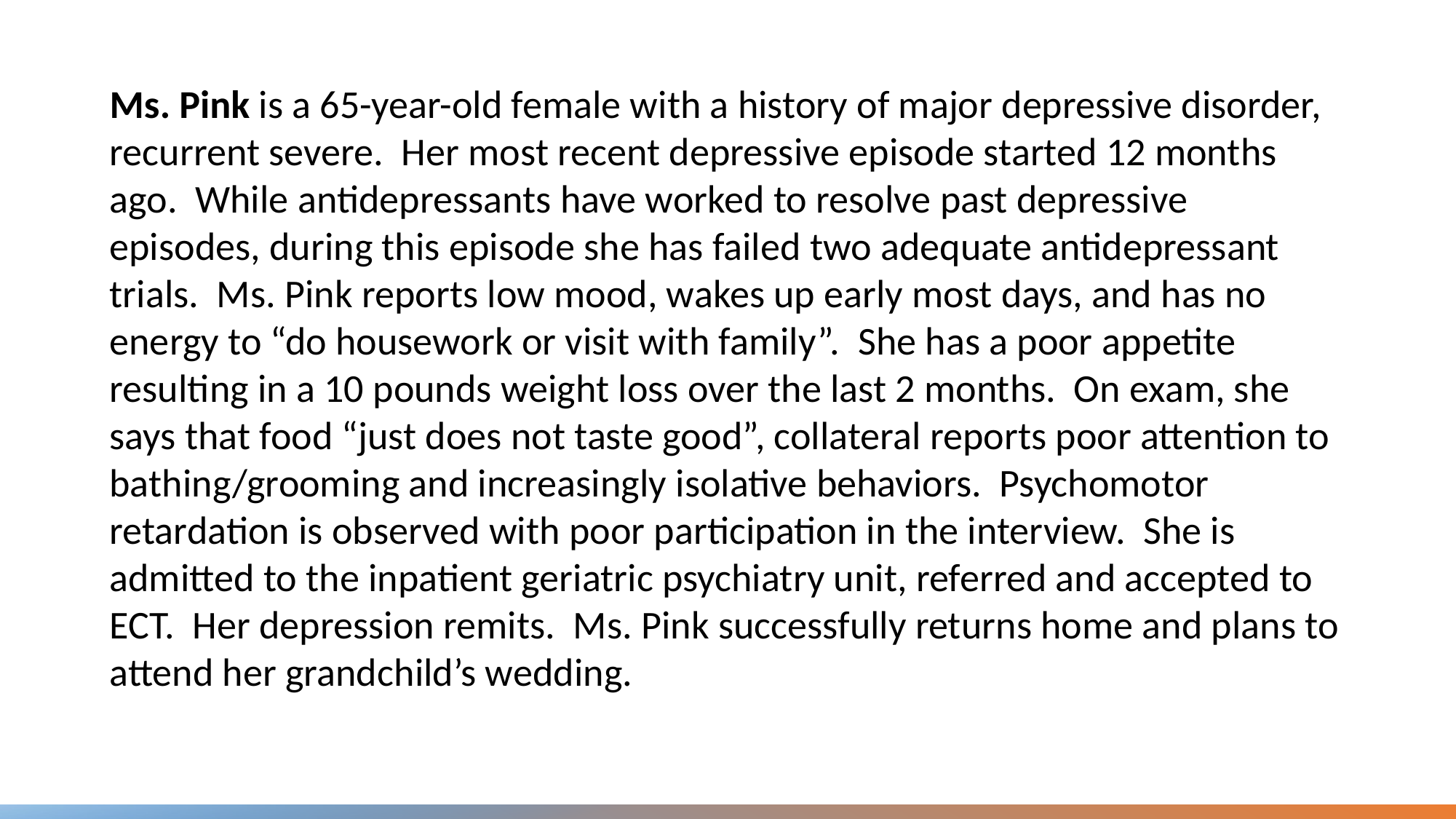

Ms. Pink is a 65-year-old female with a history of major depressive disorder, recurrent severe. Her most recent depressive episode started 12 months ago. While antidepressants have worked to resolve past depressive episodes, during this episode she has failed two adequate antidepressant trials. Ms. Pink reports low mood, wakes up early most days, and has no energy to “do housework or visit with family”. She has a poor appetite resulting in a 10 pounds weight loss over the last 2 months. On exam, she says that food “just does not taste good”, collateral reports poor attention to bathing/grooming and increasingly isolative behaviors. Psychomotor retardation is observed with poor participation in the interview. She is admitted to the inpatient geriatric psychiatry unit, referred and accepted to ECT. Her depression remits. Ms. Pink successfully returns home and plans to attend her grandchild’s wedding.

## Slide 7
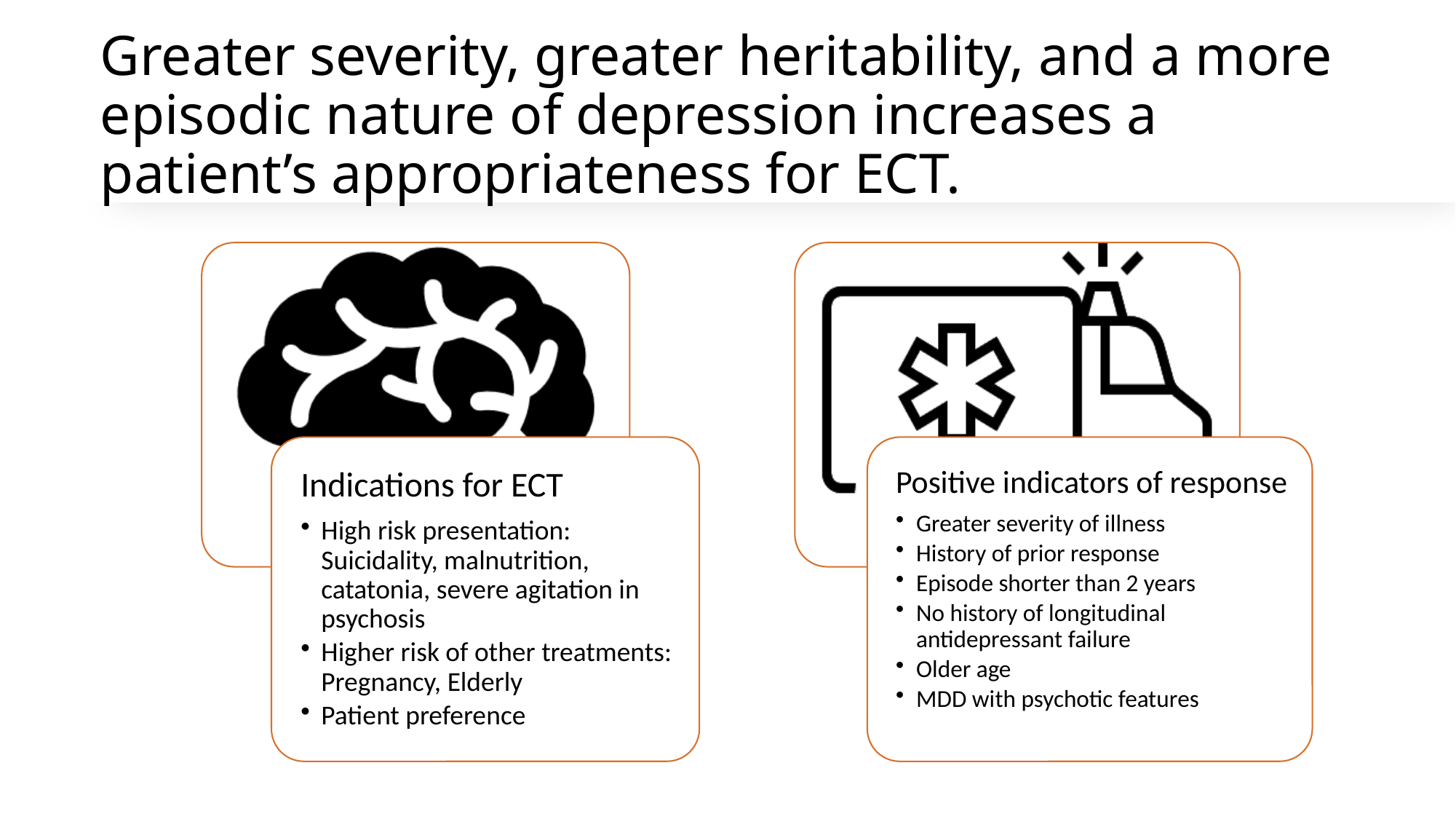

# Greater severity, greater heritability, and a more episodic nature of depression increases a patient’s appropriateness for ECT.

## Slide 8
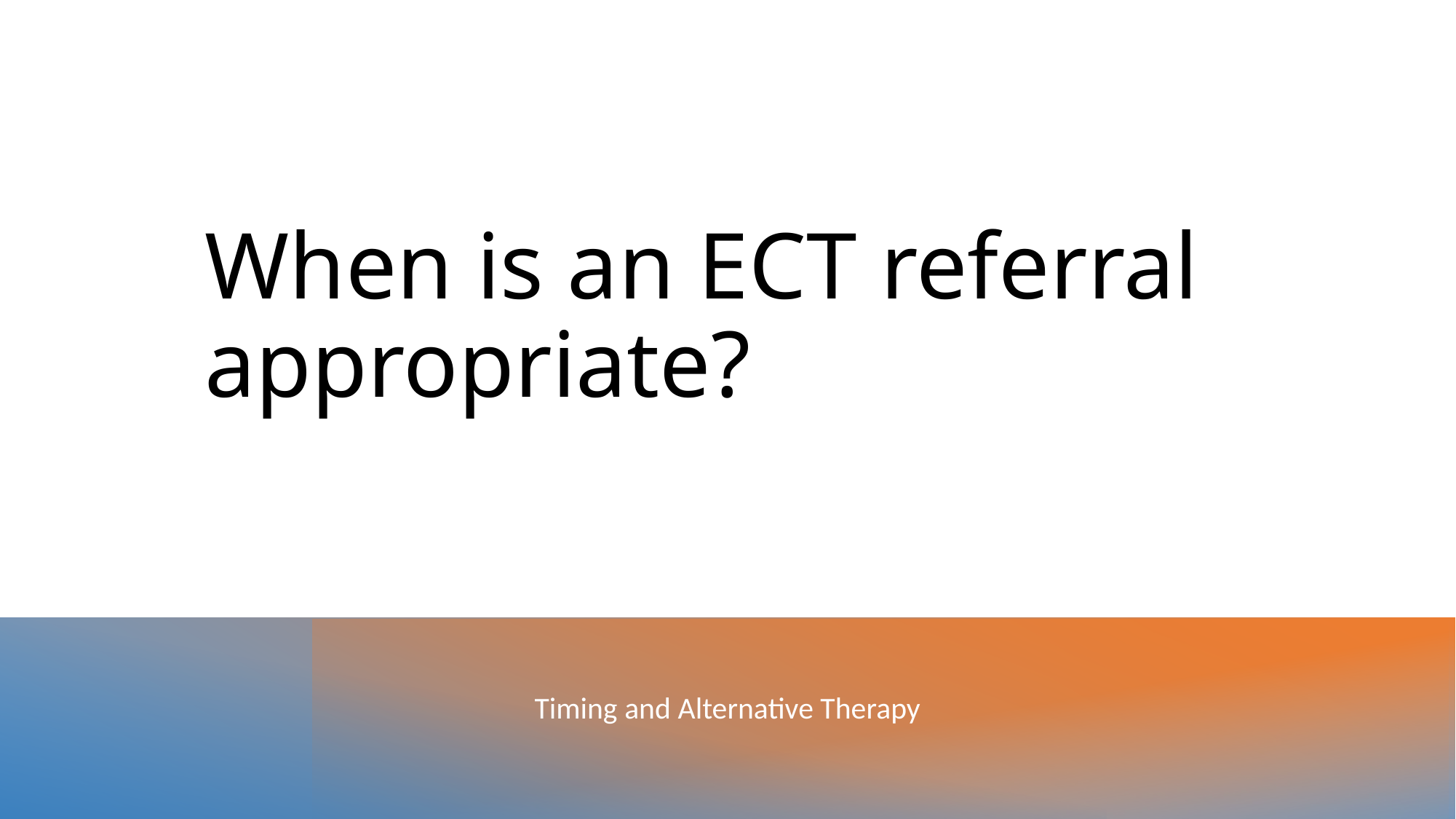

# When is an ECT referral appropriate?
Timing and Alternative Therapy

## Slide 9
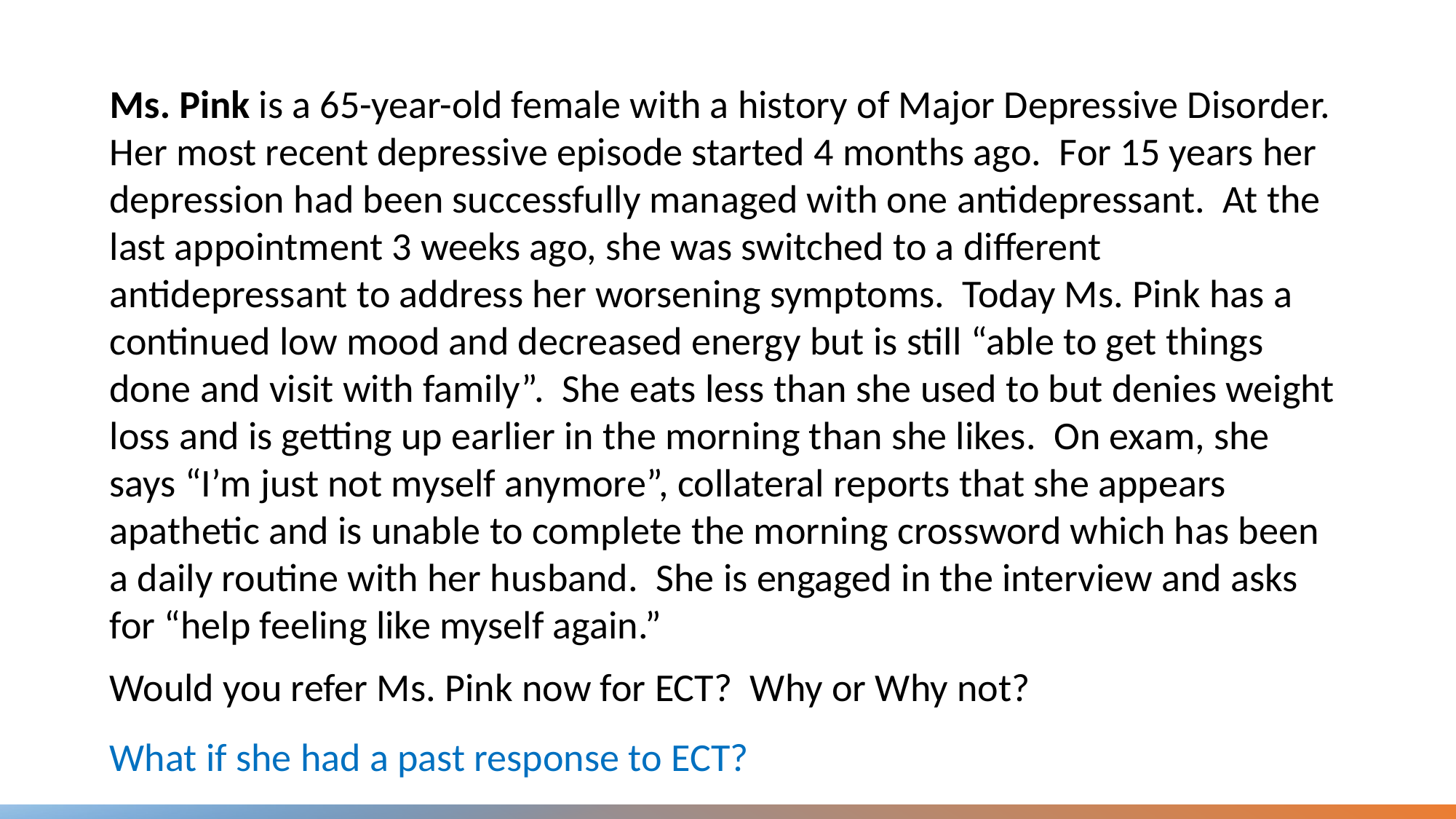

Ms. Pink is a 65-year-old female with a history of Major Depressive Disorder. Her most recent depressive episode started 4 months ago. For 15 years her depression had been successfully managed with one antidepressant. At the last appointment 3 weeks ago, she was switched to a different antidepressant to address her worsening symptoms. Today Ms. Pink has a continued low mood and decreased energy but is still “able to get things done and visit with family”. She eats less than she used to but denies weight loss and is getting up earlier in the morning than she likes. On exam, she says “I’m just not myself anymore”, collateral reports that she appears apathetic and is unable to complete the morning crossword which has been a daily routine with her husband. She is engaged in the interview and asks for “help feeling like myself again.”
Would you refer Ms. Pink now for ECT? Why or Why not?
What if she had a past response to ECT?

## Slide 10
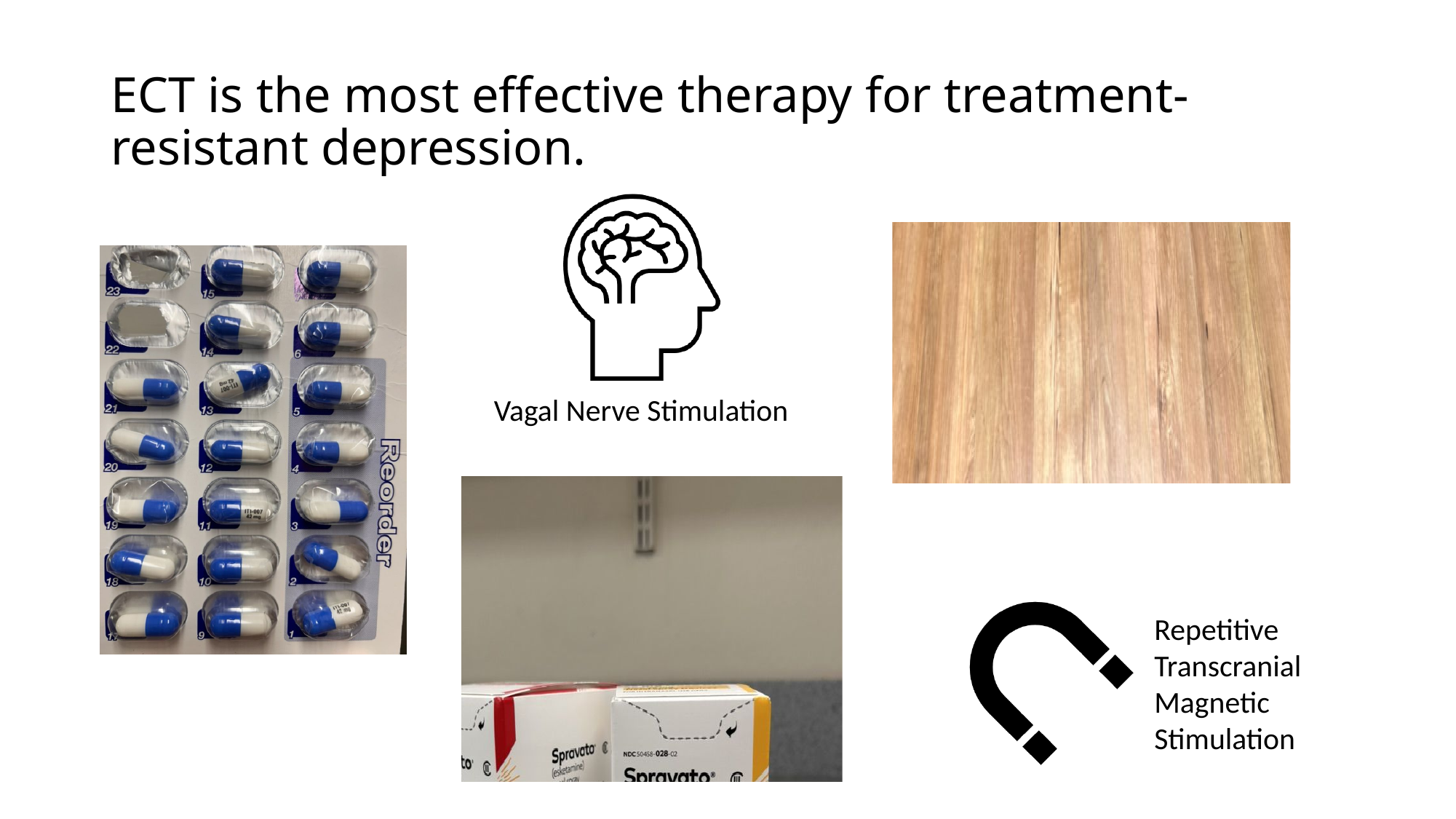

# ECT is the most effective therapy for treatment-resistant depression.
Vagal Nerve Stimulation
Repetitive Transcranial Magnetic Stimulation

## Slide 11
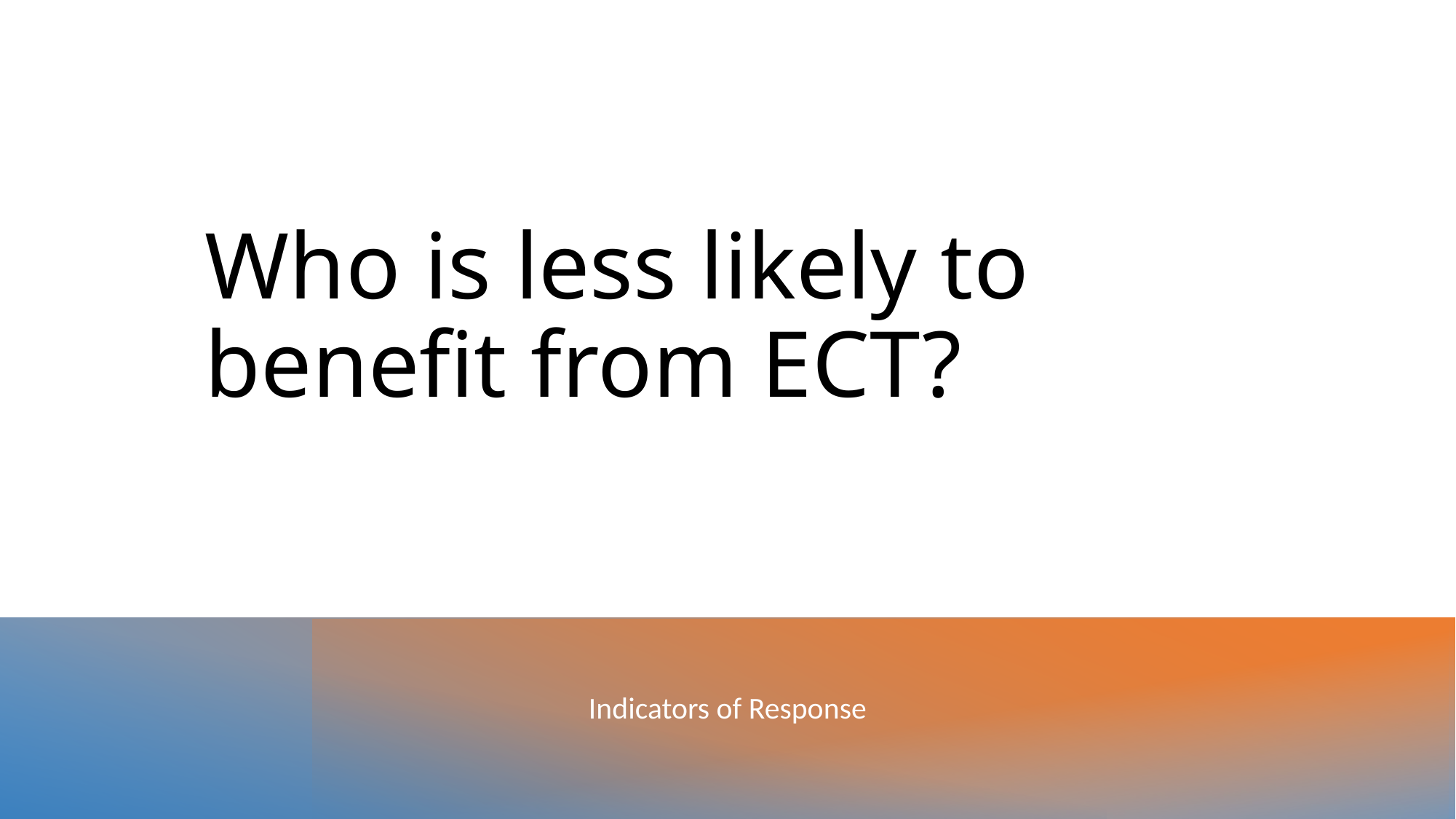

# Who is less likely to benefit from ECT?
Indicators of Response

## Slide 12
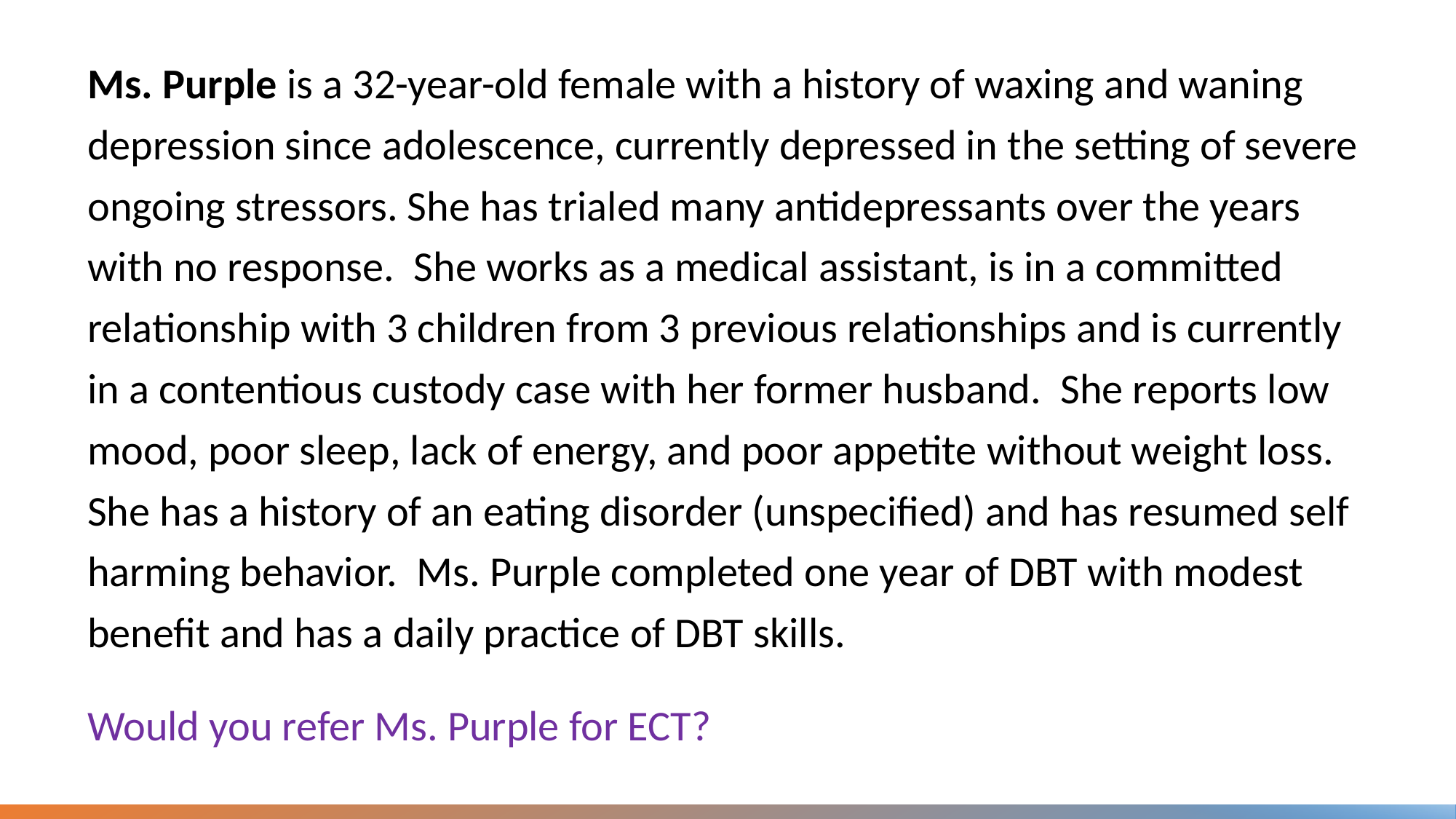

Ms. Purple is a 32-year-old female with a history of waxing and waning depression since adolescence, currently depressed in the setting of severe ongoing stressors. She has trialed many antidepressants over the years with no response. She works as a medical assistant, is in a committed relationship with 3 children from 3 previous relationships and is currently in a contentious custody case with her former husband. She reports low mood, poor sleep, lack of energy, and poor appetite without weight loss. She has a history of an eating disorder (unspecified) and has resumed self harming behavior. Ms. Purple completed one year of DBT with modest benefit and has a daily practice of DBT skills.
Would you refer Ms. Purple for ECT?

## Slide 13
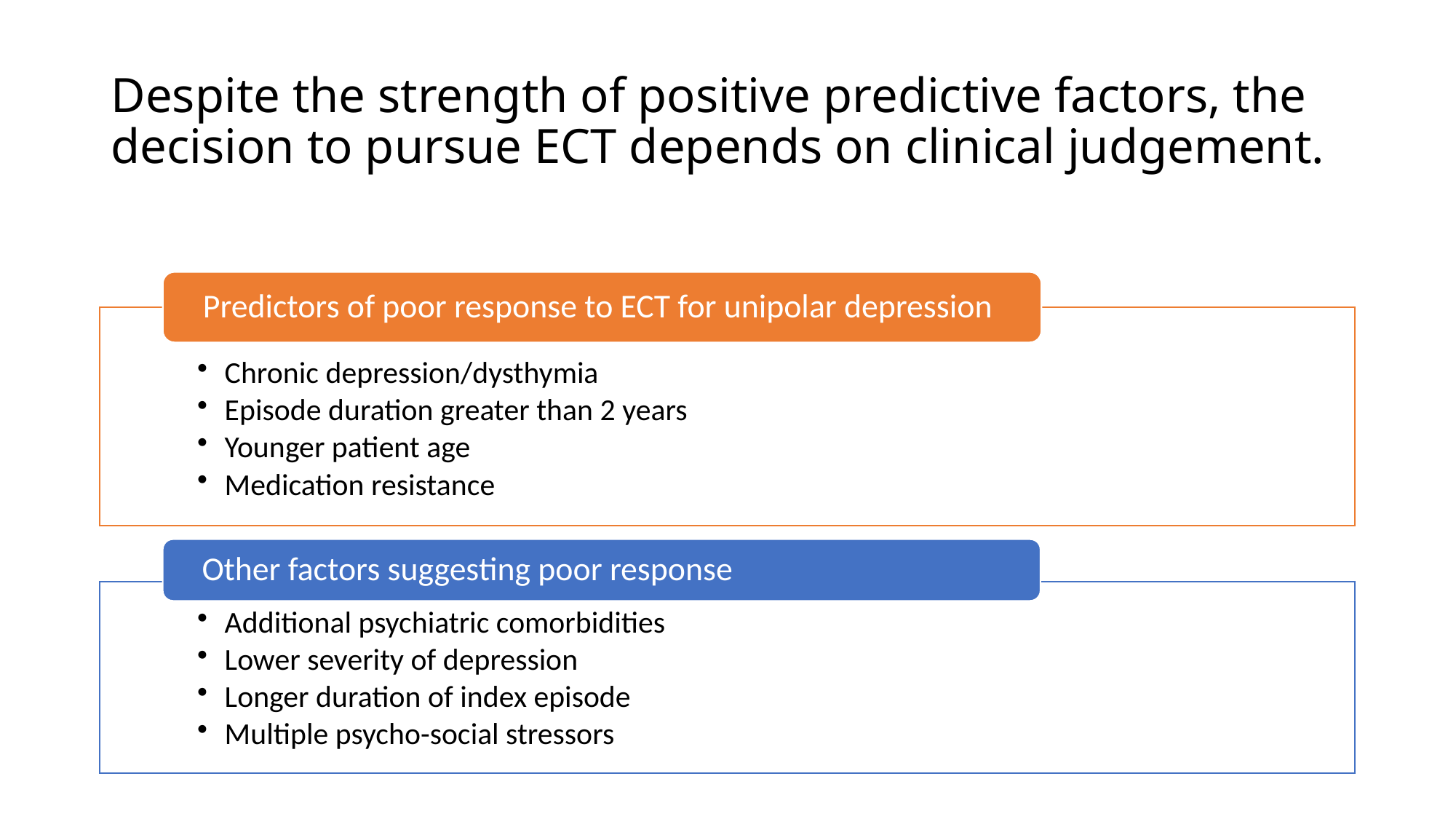

# Despite the strength of positive predictive factors, the decision to pursue ECT depends on clinical judgement.

## Slide 14
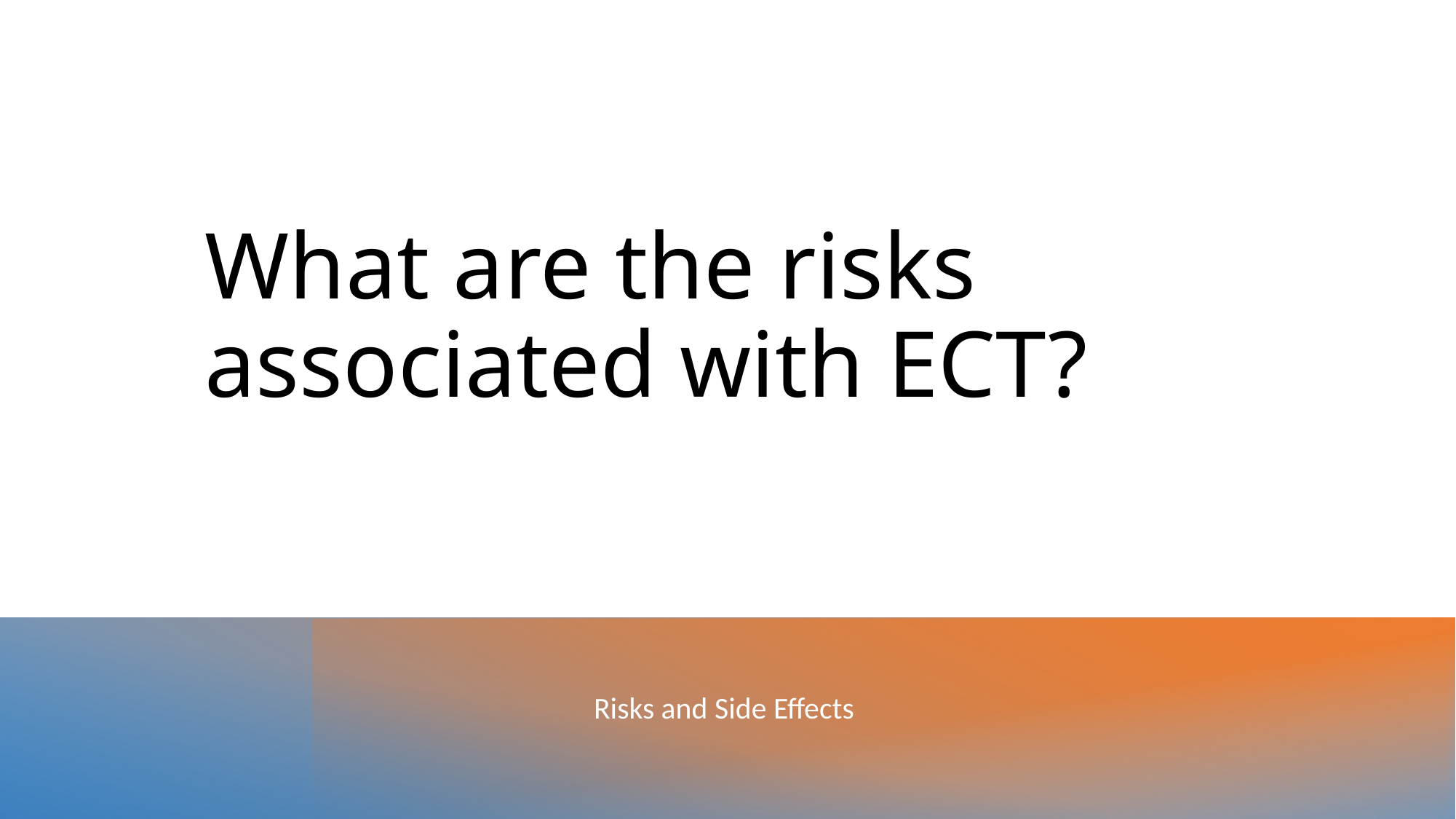

# What are the risks associated with ECT?
Risks and Side Effects

## Slide 15
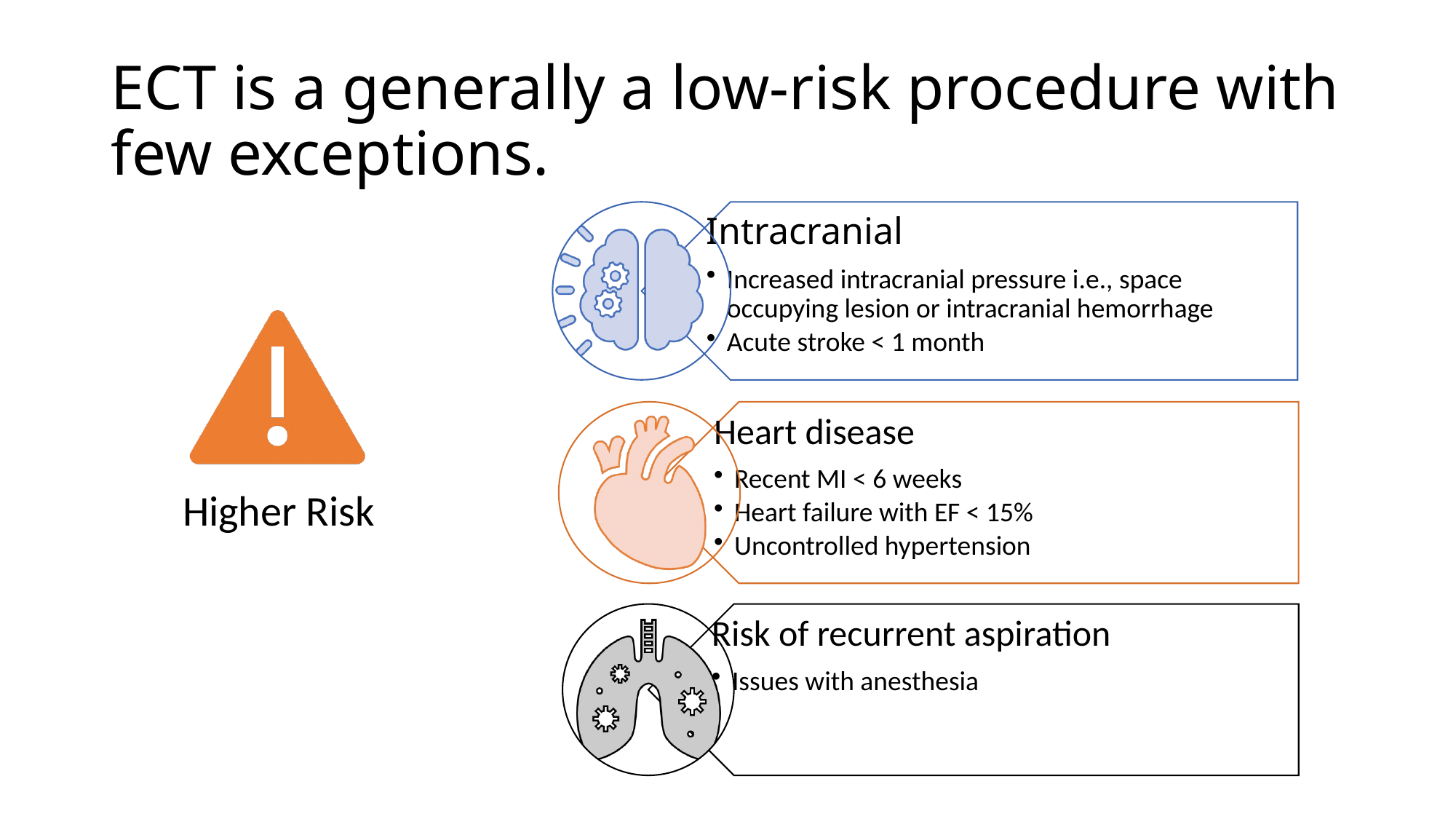

# ECT is a generally a low-risk procedure with few exceptions.
Higher Risk

## Slide 16
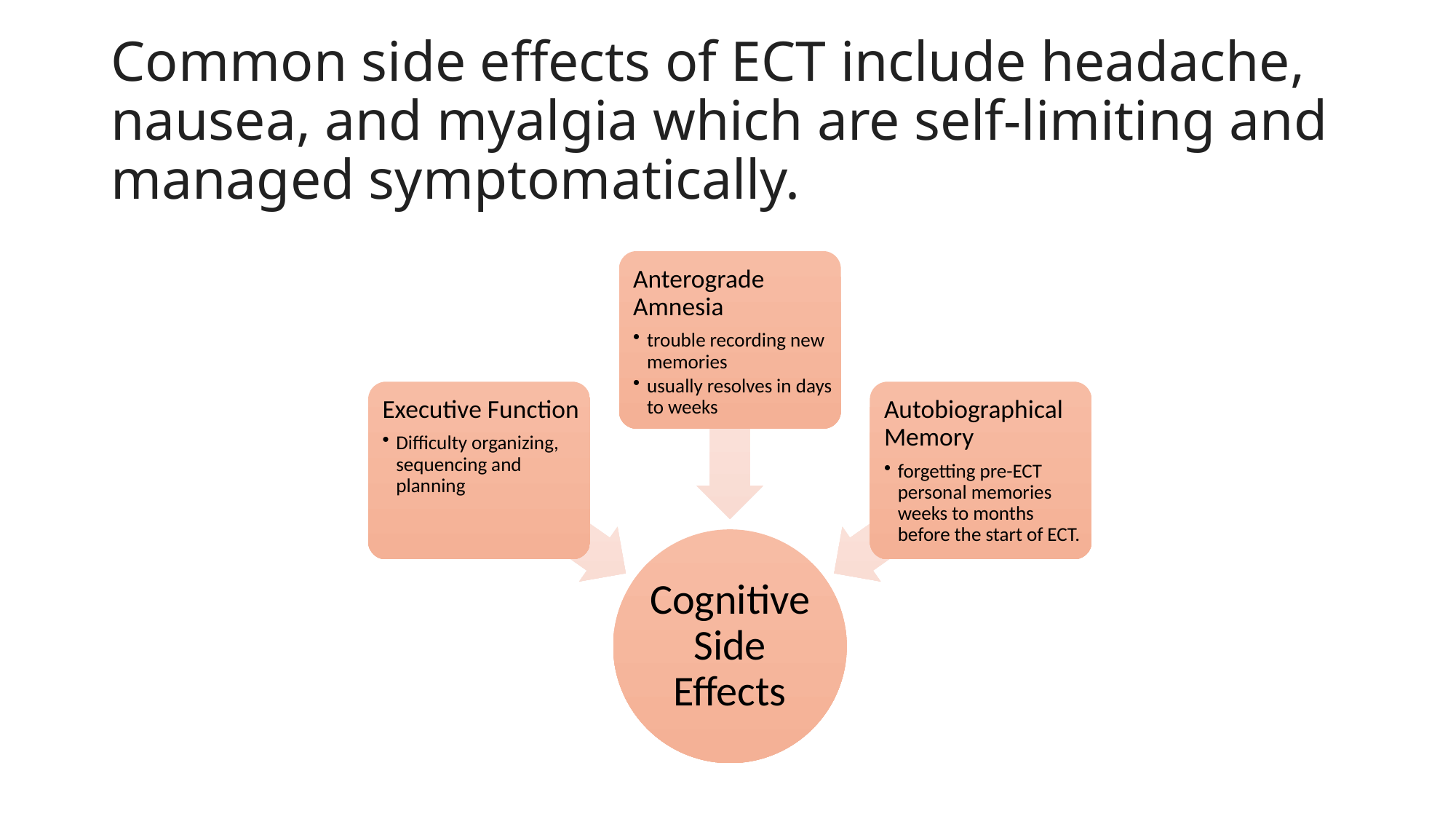

# Common side effects of ECT include headache, nausea, and myalgia which are self-limiting and managed symptomatically.

## Slide 17
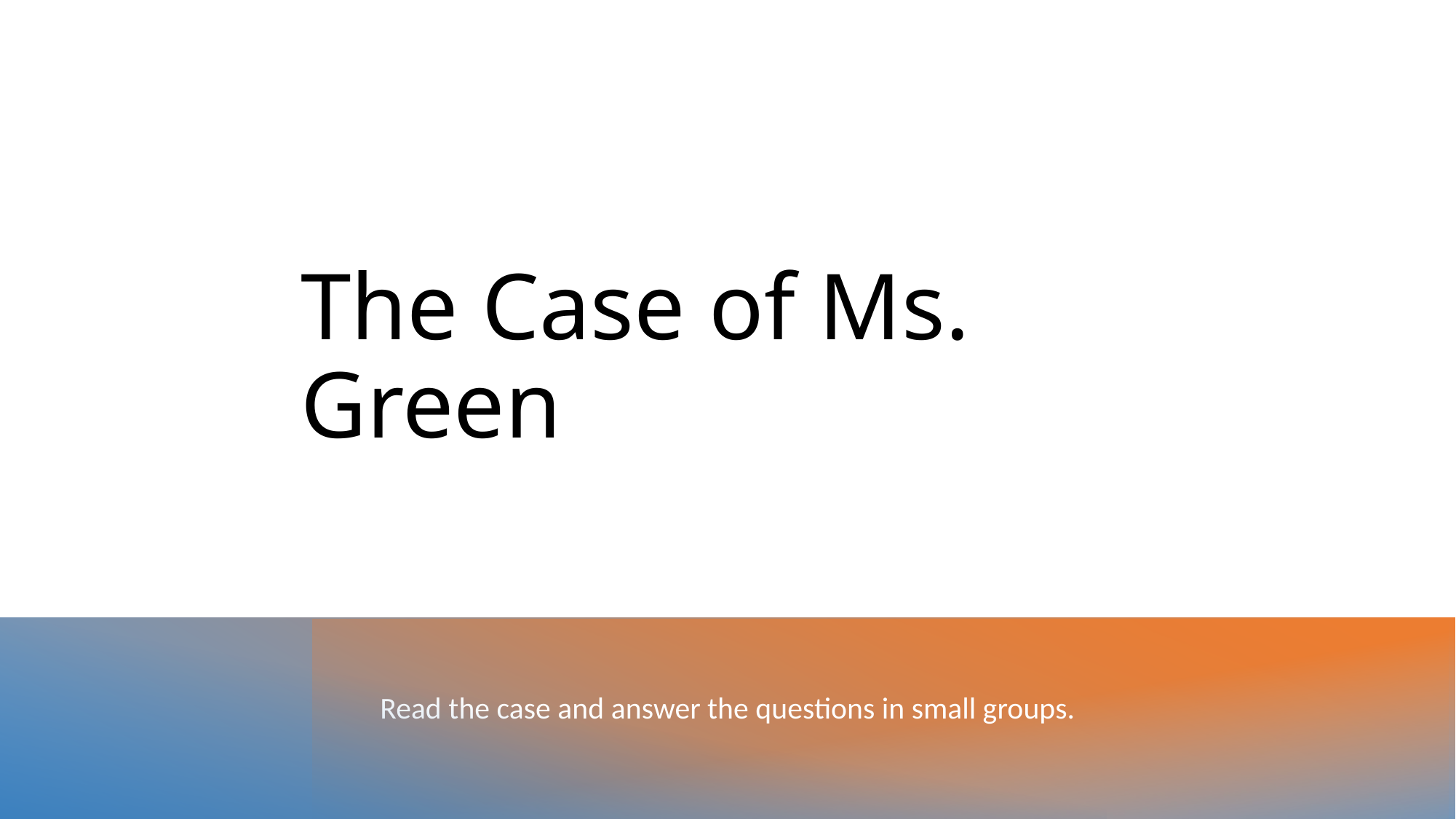

# The Case of Ms. Green
Read the case and answer the questions in small groups.

## Slide 18
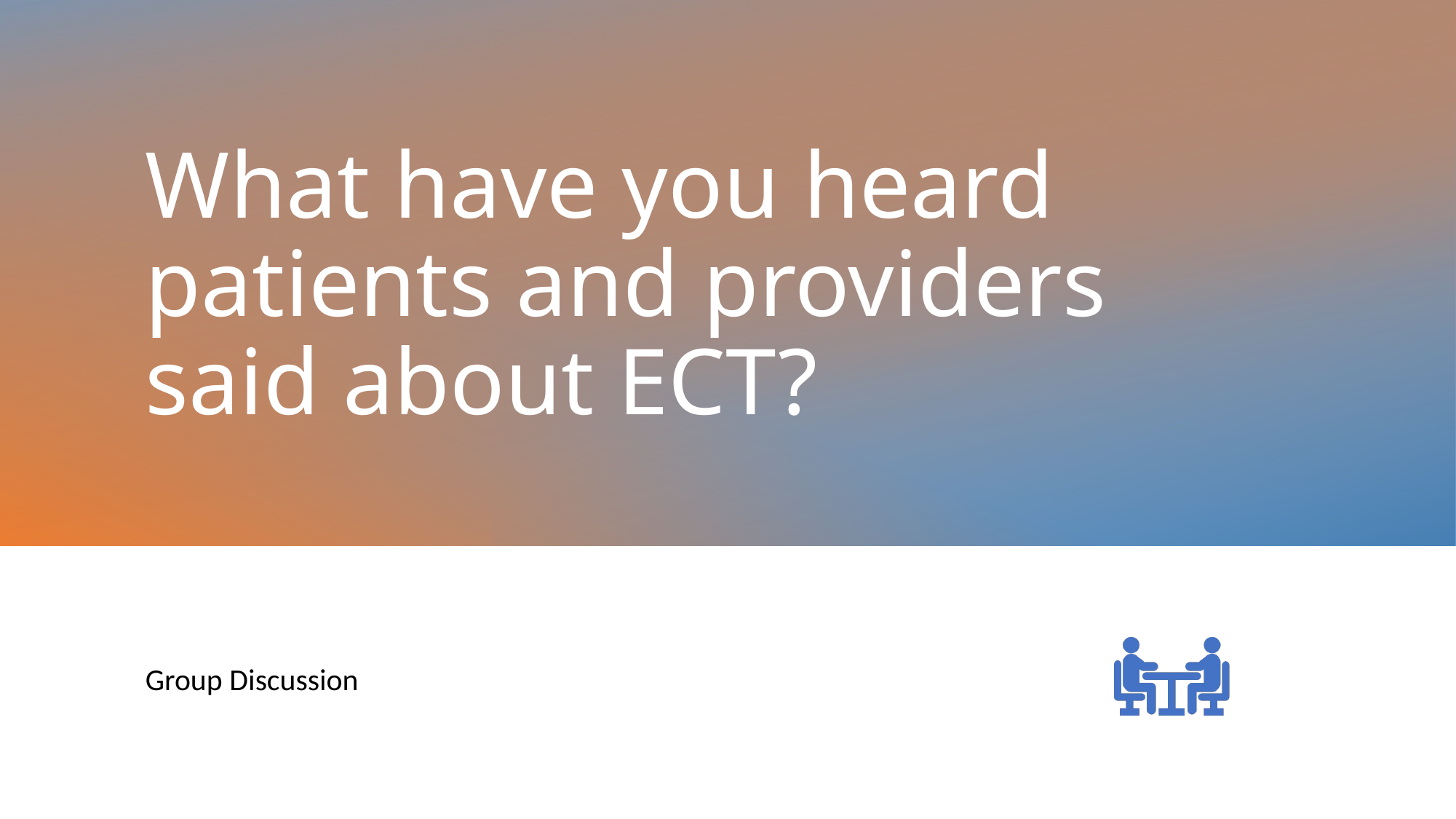

# What have you heard patients and providers said about ECT?
Group Discussion

## Slide 19
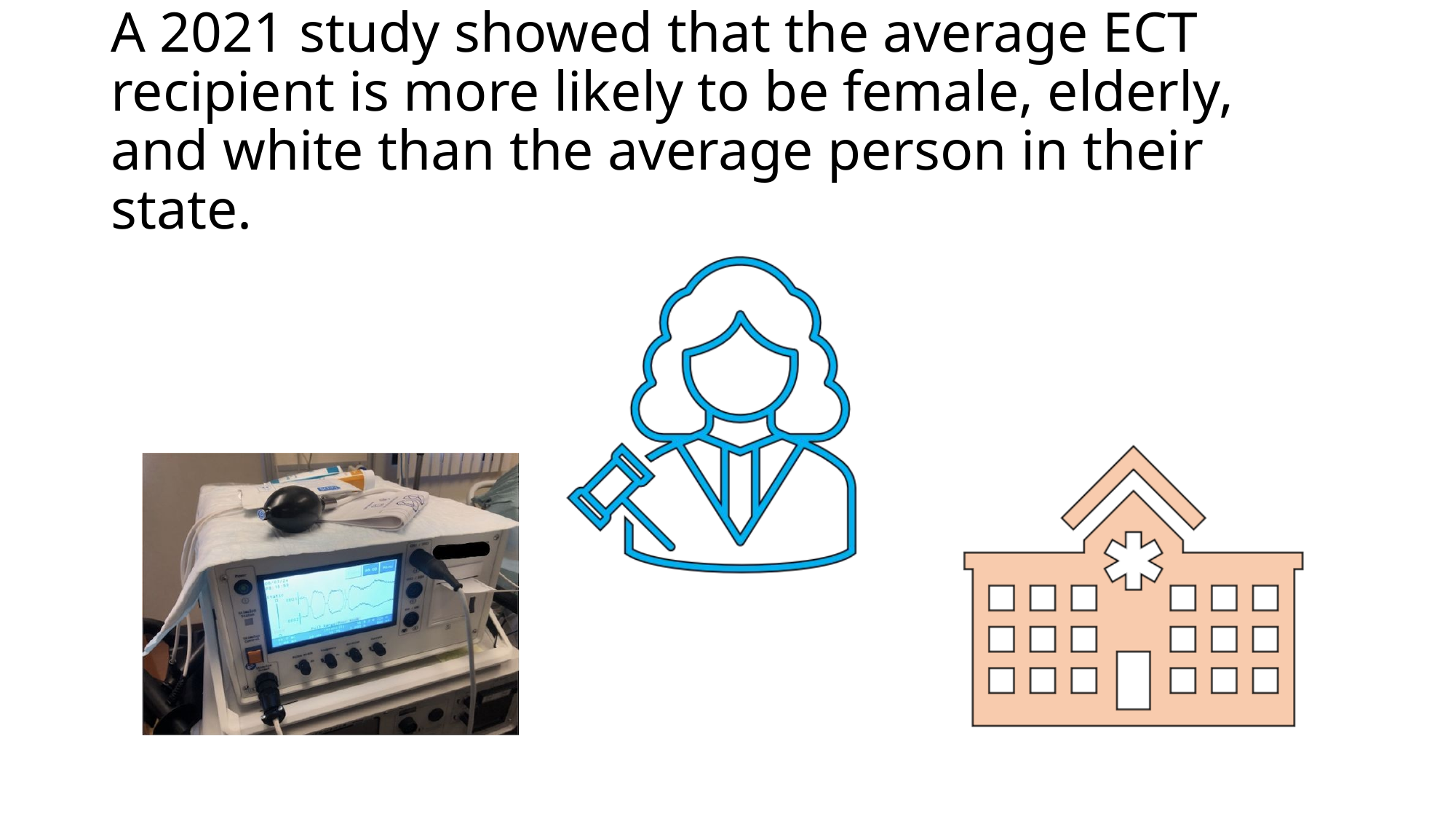

# A 2021 study showed that the average ECT recipient is more likely to be female, elderly, and white than the average person in their state.

## Slide 20
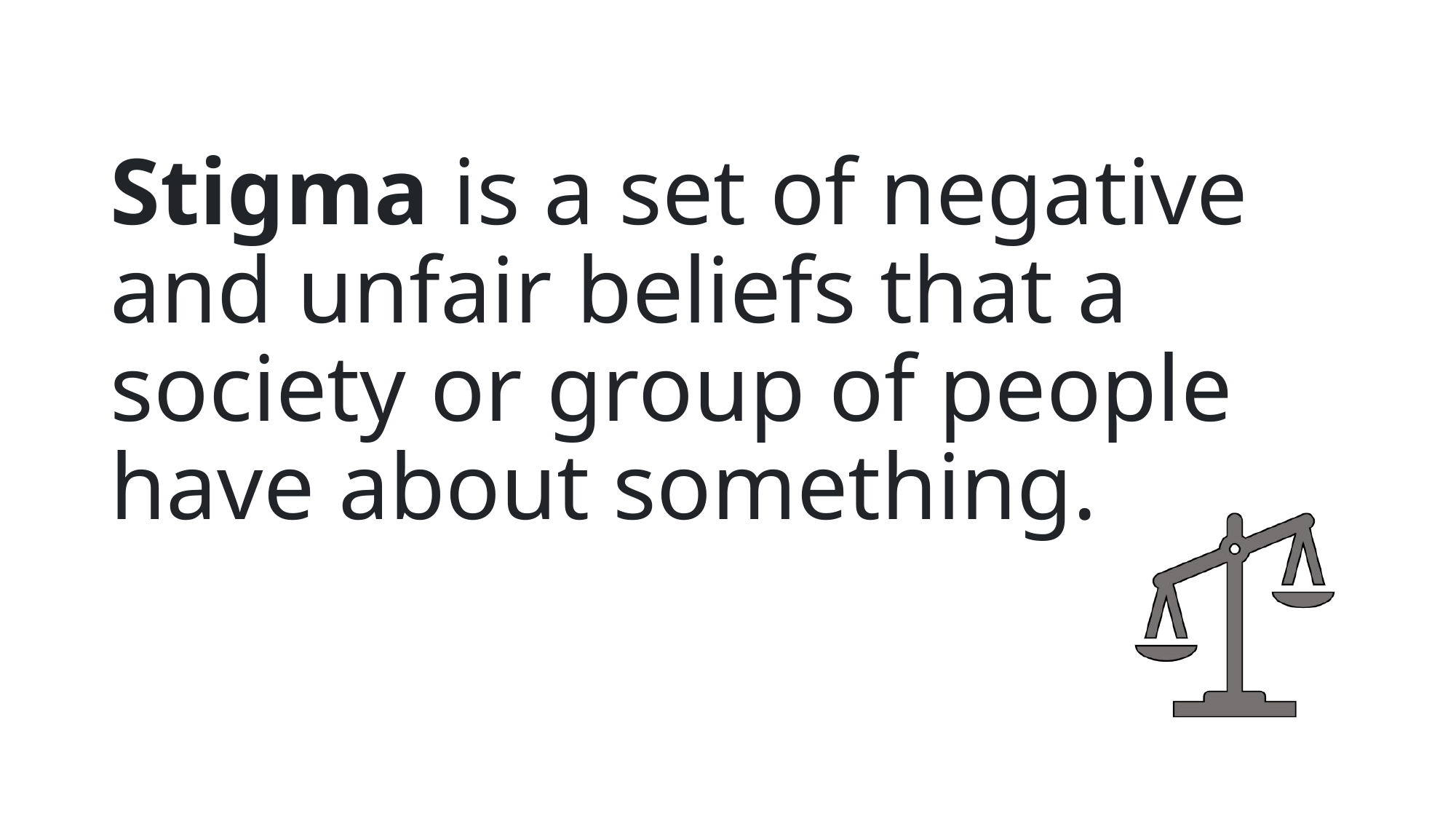

# Stigma is a set of negative and unfair beliefs that a society or group of people have about something.

## Slide 21
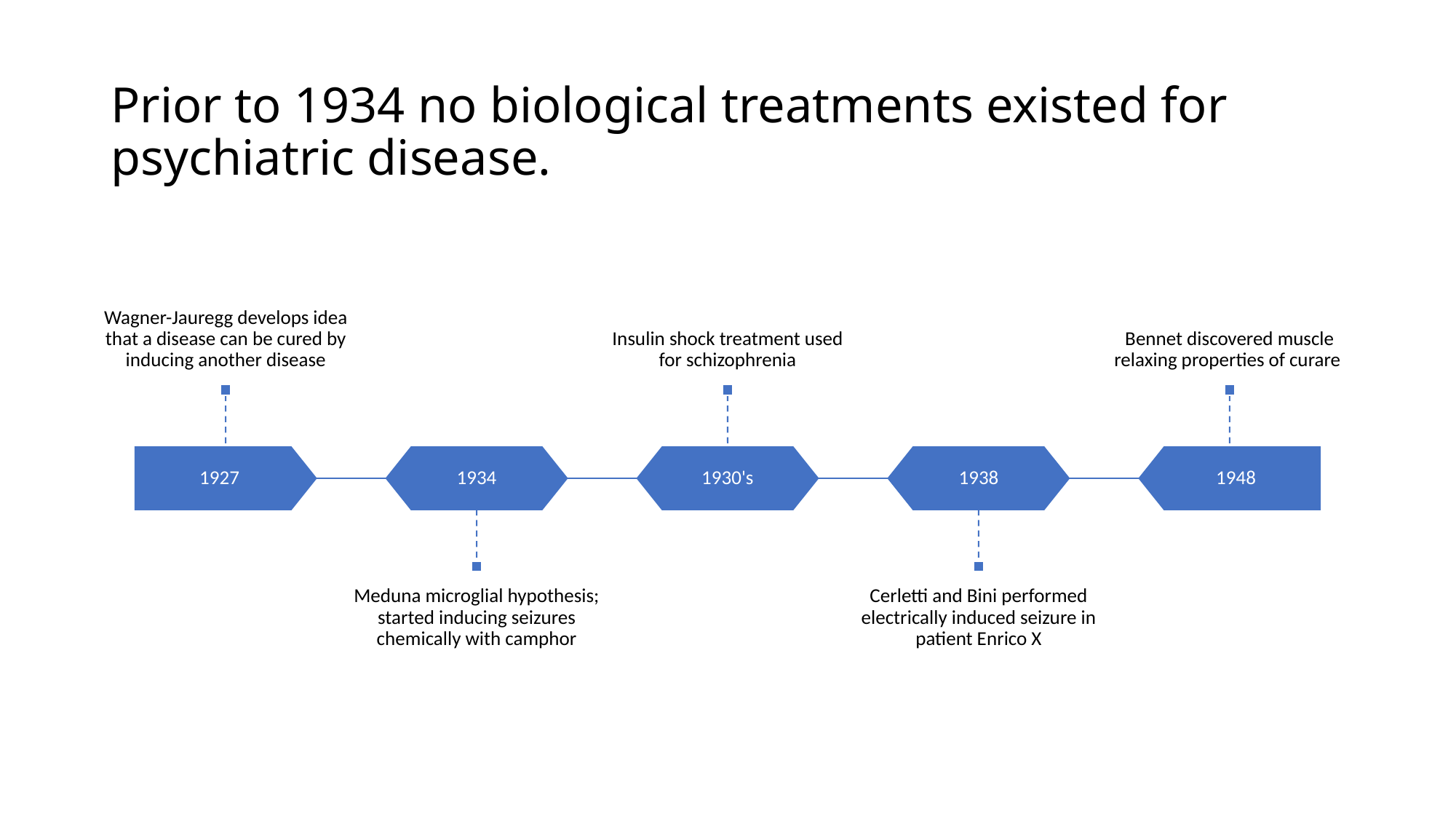

# Prior to 1934 no biological treatments existed for psychiatric disease.

## Slide 22
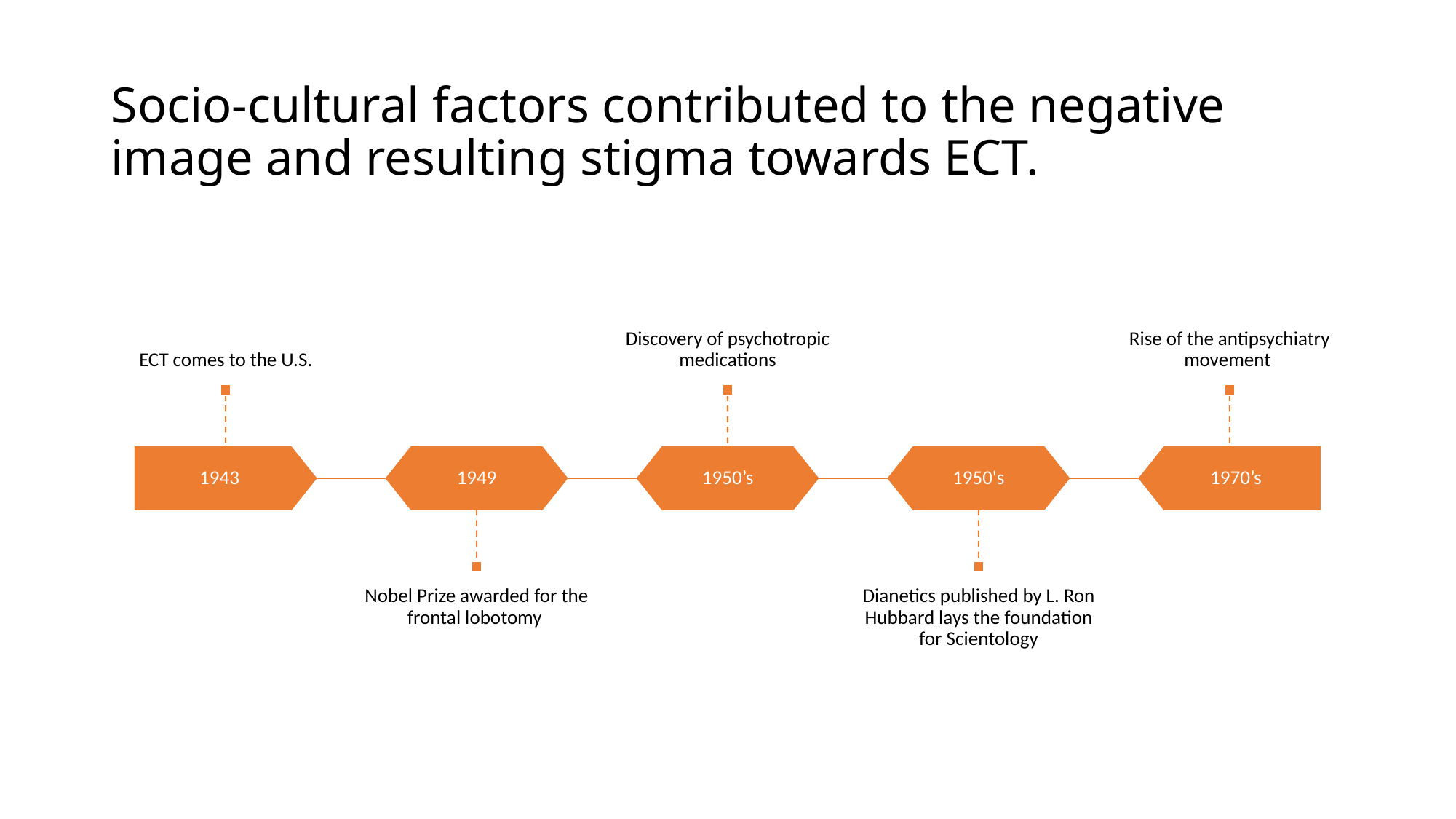

# Socio-cultural factors contributed to the negative image and resulting stigma towards ECT.

## Slide 23
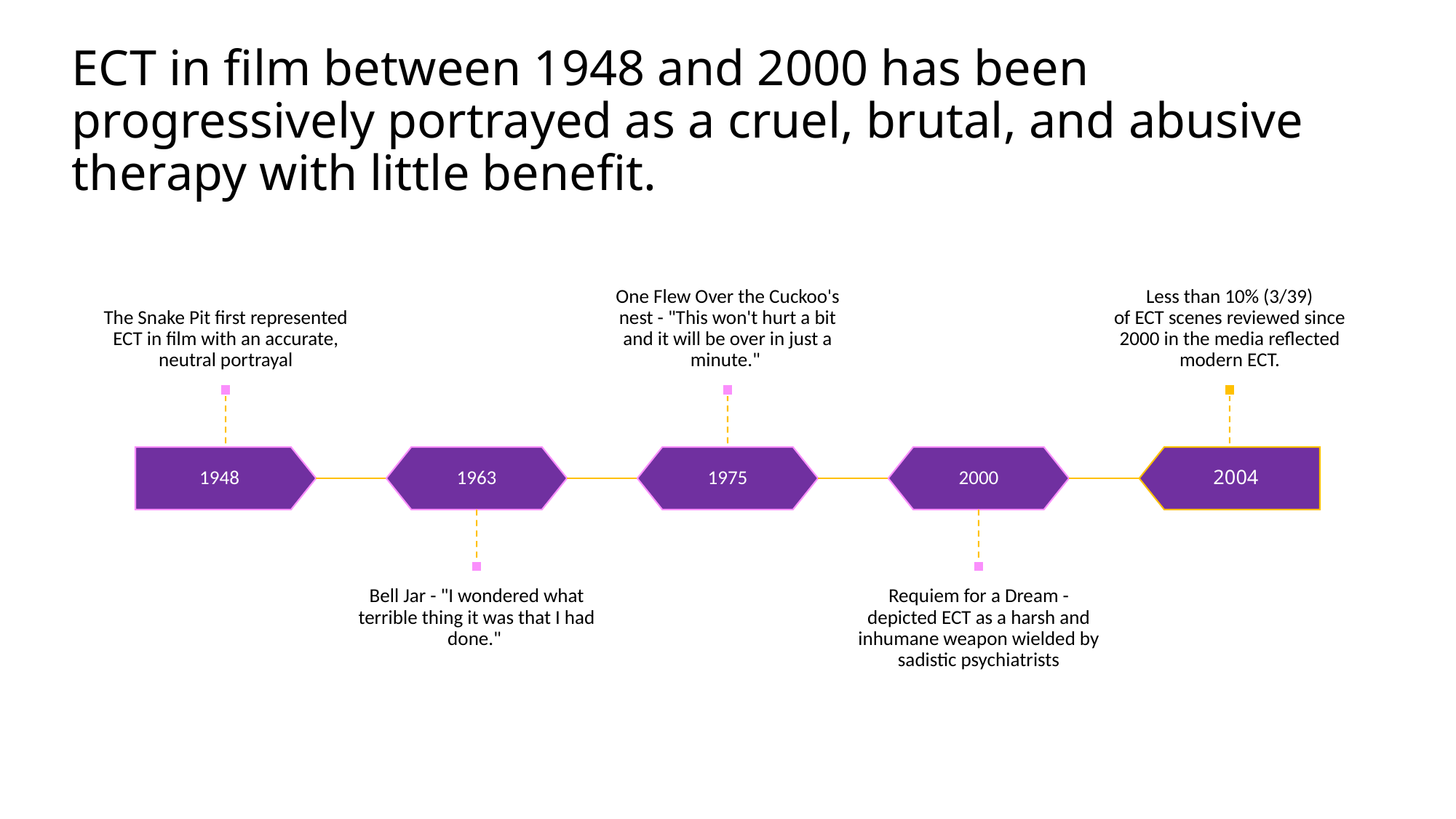

# ECT in film between 1948 and 2000 has been progressively portrayed as a cruel, brutal, and abusive therapy with little benefit.

## Slide 24
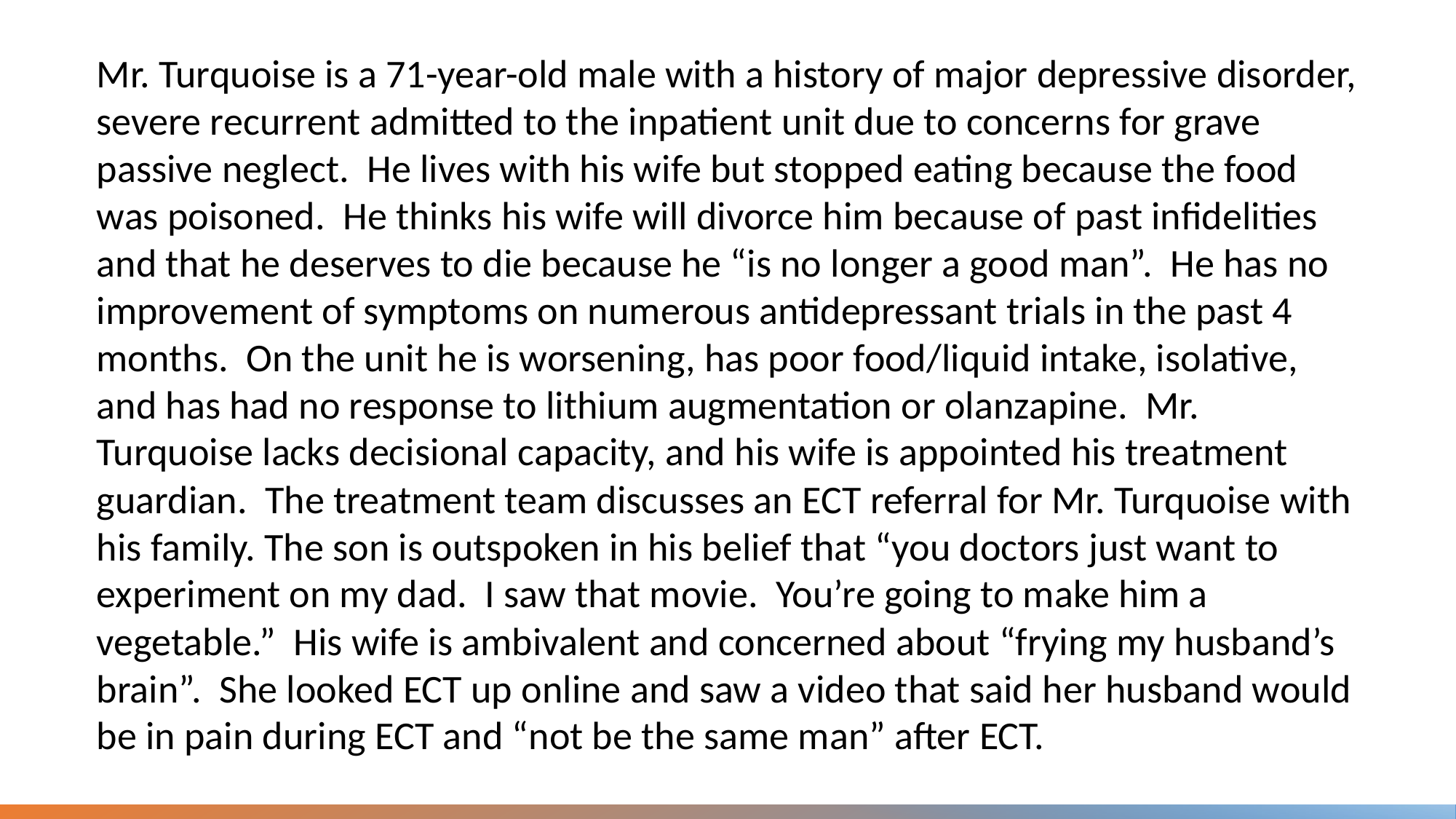

Mr. Turquoise is a 71-year-old male with a history of major depressive disorder, severe recurrent admitted to the inpatient unit due to concerns for grave passive neglect. He lives with his wife but stopped eating because the food was poisoned. He thinks his wife will divorce him because of past infidelities and that he deserves to die because he “is no longer a good man”. He has no improvement of symptoms on numerous antidepressant trials in the past 4 months. On the unit he is worsening, has poor food/liquid intake, isolative, and has had no response to lithium augmentation or olanzapine. Mr. Turquoise lacks decisional capacity, and his wife is appointed his treatment guardian. The treatment team discusses an ECT referral for Mr. Turquoise with his family. The son is outspoken in his belief that “you doctors just want to experiment on my dad. I saw that movie. You’re going to make him a vegetable.” His wife is ambivalent and concerned about “frying my husband’s brain”. She looked ECT up online and saw a video that said her husband would be in pain during ECT and “not be the same man” after ECT.

## Slide 25
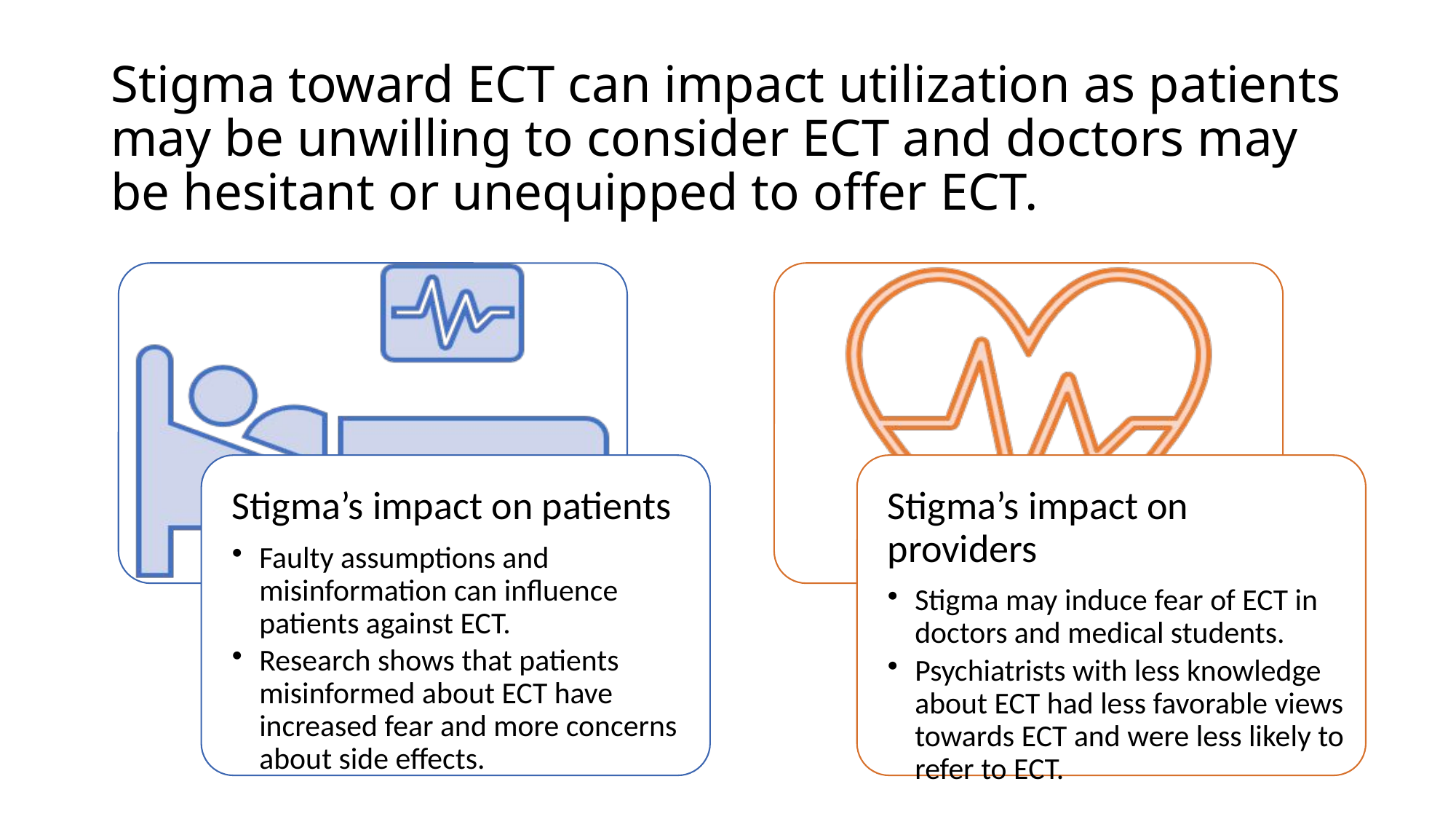

# Stigma toward ECT can impact utilization as patients may be unwilling to consider ECT and doctors may be hesitant or unequipped to offer ECT.

## Slide 26
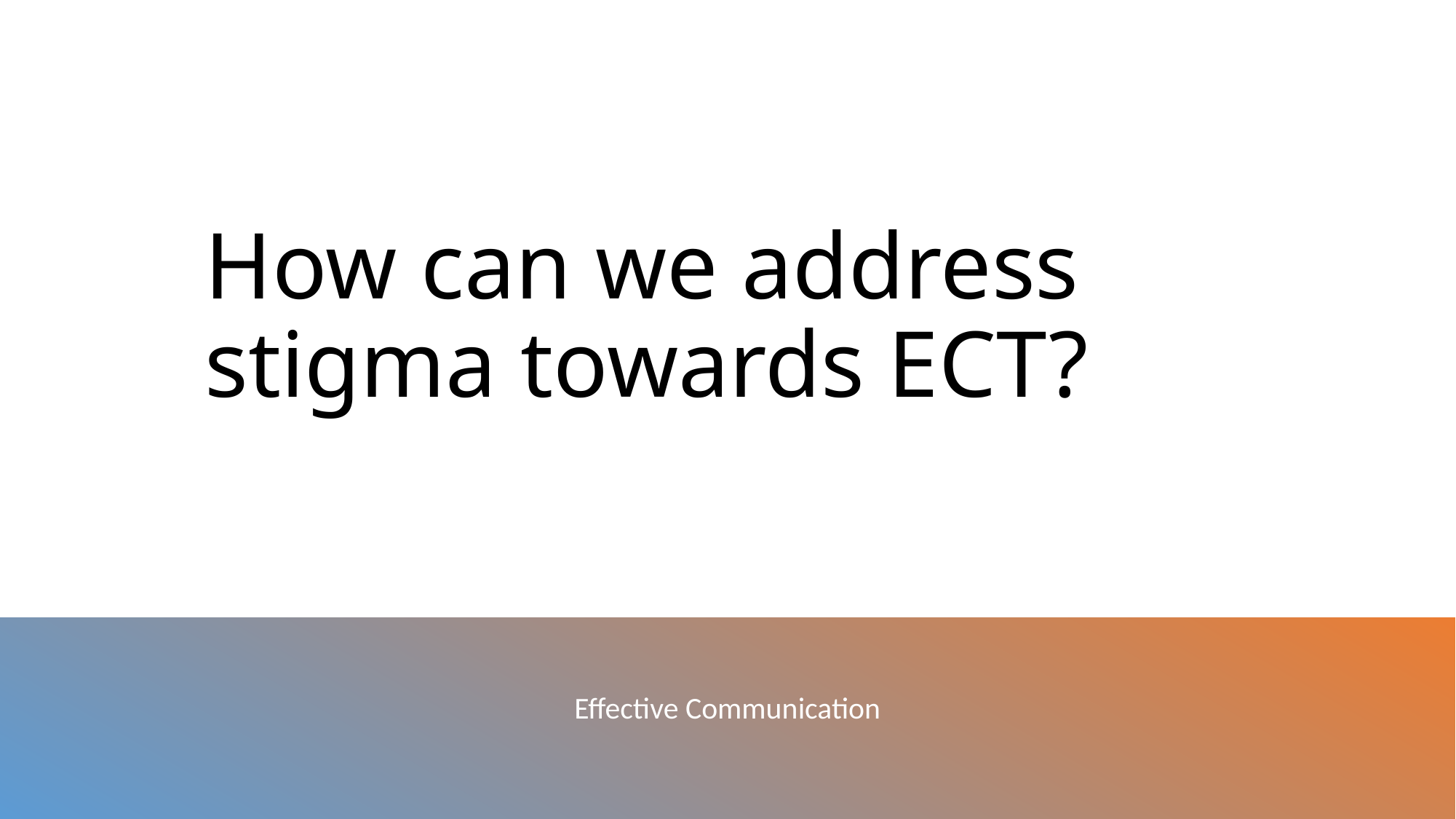

# How can we address stigma towards ECT?
Effective Communication

## Slide 27
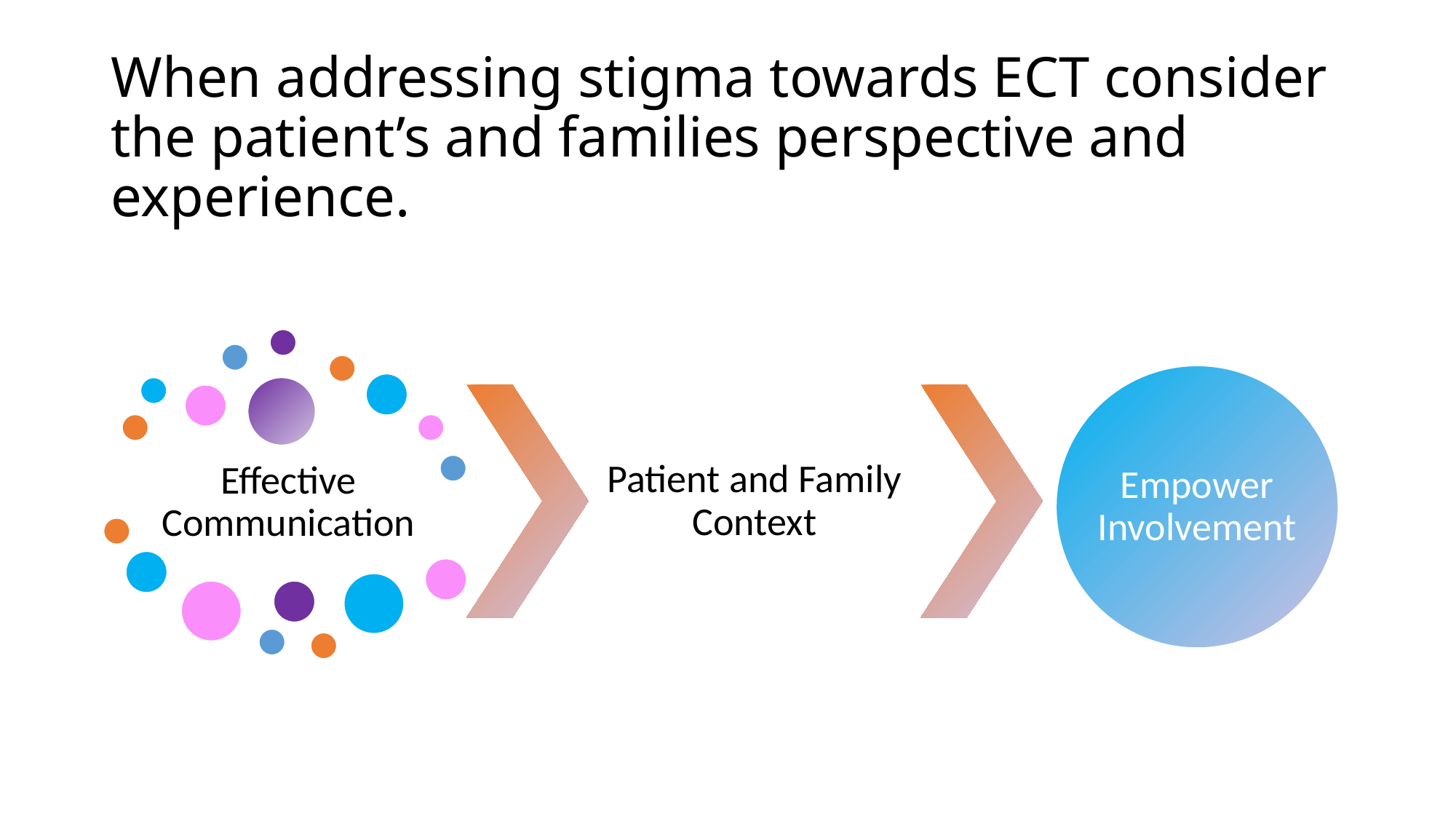

# When addressing stigma towards ECT consider the patient’s and families perspective and experience.

## Slide 28
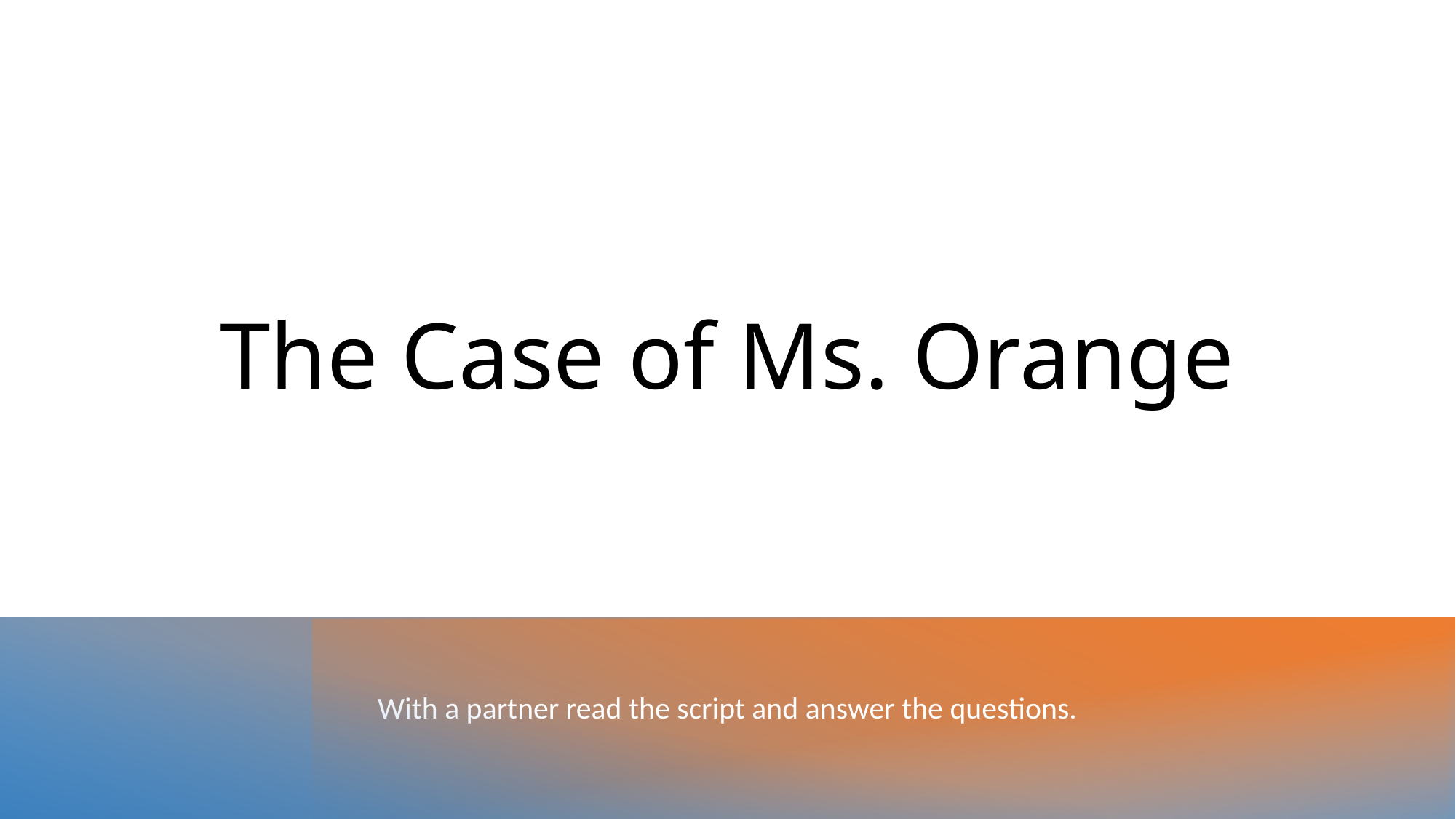

# The Case of Ms. Orange
With a partner read the script and answer the questions.

## Slide 29
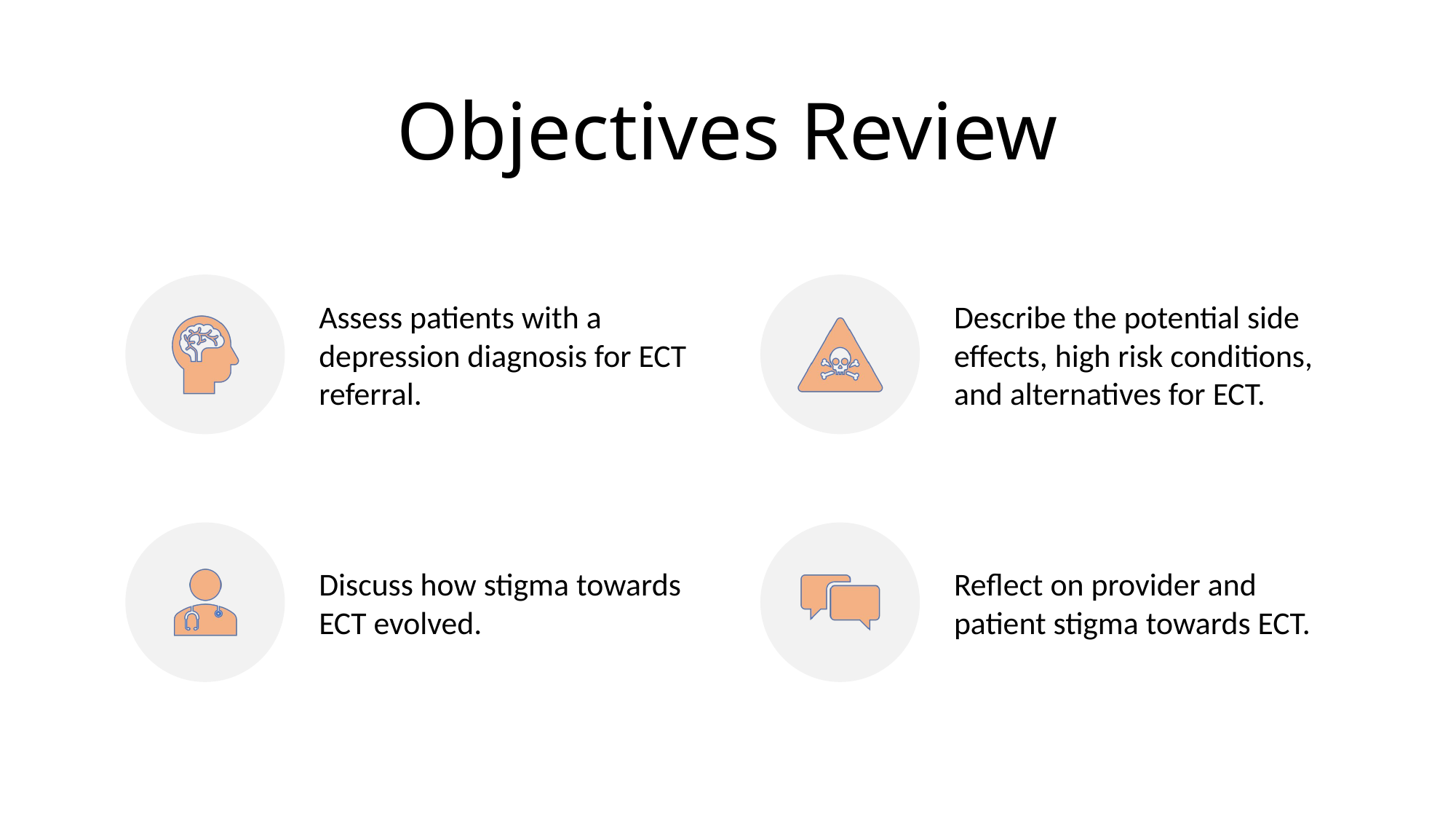

# Objectives Review

## Slide 30
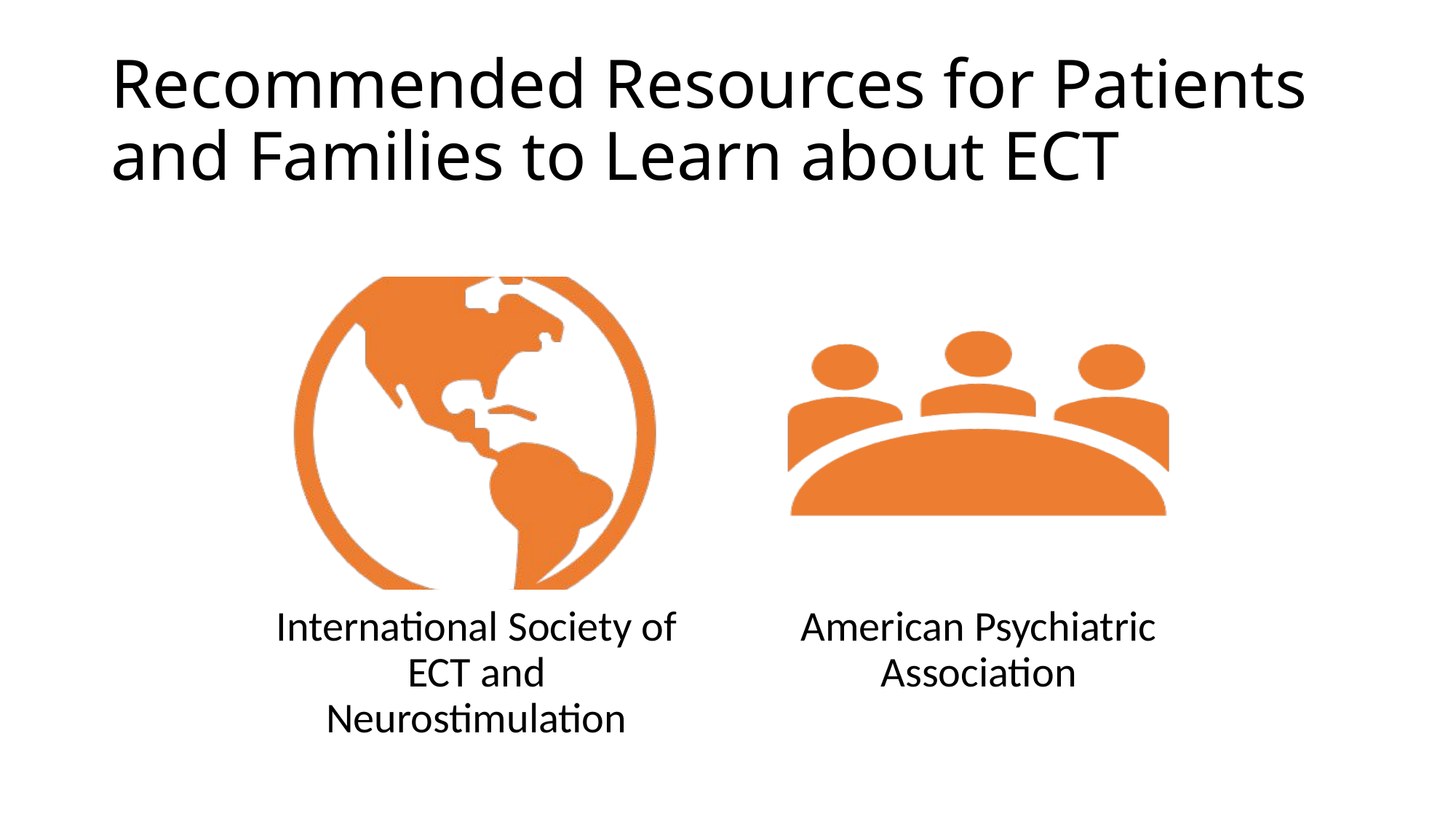

# Recommended Resources for Patients and Families to Learn about ECT

## Slide 31
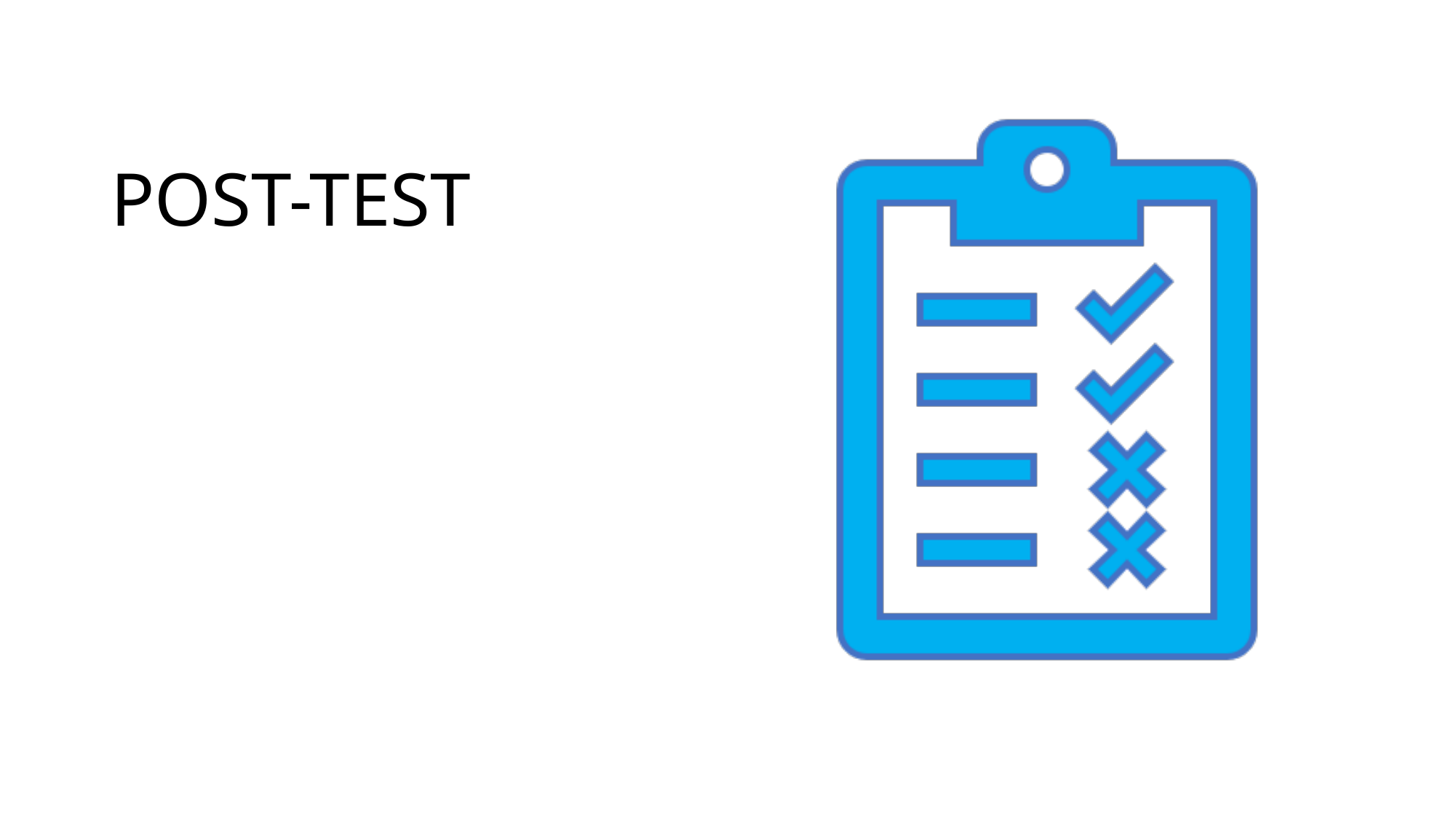

# POST-TEST
